# *ClAPRT3* ‐Mediated Adenine Salvage Pathway Enhances Purine Metabolites to Sustain Seed Vigour During Selfing in *Cunninghamia lanceolata*

**DOI:** 10.1111/pbi.70363

**Published:** 2025-09-16

**Authors:** Houyin Deng, Ye Zhao, Jie Liu, Kunjin Han, Juan Han, Meng Ke, Rong Huang, Ruping Wei, Yousry A. El‐Kassaby, Runhui Wang, Shu Yan, Yuhan Sun, Yun Li, Huiquan Zheng

**Affiliations:** ^1^ State Key Laboratory of Tree Genetics and Breeding, National Engineering Research Center of Tree Breeding and Ecological Restoration, Engineering Technology Research Center of Black Locust of National Forestry and Grassland Administration, College of Biological Sciences and Technology Beijing Forestry University Beijing People's Republic of China; ^2^ Guangdong Provincial Key Laboratory of Silviculture, Protection and Utilization Guangdong Academy of Forestry Guangzhou People's Republic of China; ^3^ Department of Forest and Conservation Sciences Faculty of Forestry The University of British Columbia Vancouver British Columbia Canada

**Keywords:** adenine phosphoribosyl transferase, adenine salvage, *Cunninghamia lanceolata*, purine metabolites, seed vigour, selfing

## Abstract

Selfing often causes inbreeding depression, especially during seed and seedling stages. However, some selfed progeny show low inbreeding depression with enhanced vigour, differing from inbred counterparts. This study investigates the molecular mechanisms maintaining seed vigour during selfing in *Cunninghamia lanceolata* . Evaluation showed that selfed seeds had medium vigour compared to other inbred and non‐inbred seeds. Transcriptome analysis revealed similar gene expression patterns in the radicles and hypocotyls of inbred seeds. GO enrichment analysis identified adenine salvage as a key pathway related to seed vigour in selfed seeds. Purine metabolites (AMP, ADP, ATP) were higher in inbred seeds with better vigour, correlating with increased APRT enzyme activity. APRT inhibition by 6‐diaminopurine had varying effects: high‐vigour seeds were significantly inhibited, low‐vigour seeds showed weaker inhibition, and selfed seeds exhibited an intermediate response. The addition of adenine and AMP partially restored seed vigour, further supporting the role of adenine salvage in vigour maintenance. A significant positive correlation (*r* > 0.922, *p* < 0.0001) between *ClAPRT3* expression and APRT activity suggests that *ClAPRT3* reflects APRT activity. Consistently, *ClAPRT3* overexpression in *Arabidopsis thaliana* significantly enhanced radicle and hypocotyl length. Although *ClMYB23* negatively regulates *ClAPRT3* expression, no mutation was detected in the MYB binding motif among inbred progeny. Instead, variations in the ClAPRT3 coding sequence led to differences in binding energy with its ligand, which may partially explain the observed differences in APRT activity. These findings provide insights into the transcriptional and metabolic regulation of adenine salvage in maintaining seed vigour under inbreeding.

## 1 Introduction

Selfing, an extreme form of inbreeding, often results in reduced seed quality and quantity, lower seedling establishment and survival rates, and increased mortality, collectively referred to as inbreeding depression (Charlesworth and Willis 2009). Despite these negative effects, selfing is a vital reproductive strategy in plants, occurring in 10% to 15% of flowering plants and playing a key role in approximately 40% of species with mixed mating systems (Goodwillie et al. 2005; Wright et al. 2013; van Ginkel and Flipphi 2020). Selfing provides the advantage of reproductive assurance, ensuring the production of viable seeds and the continued species survival (Herlihy and Eckert 2002; Zhang et al. 2011; Rymer et al. 2015). Additionally, selfing promotes genetic purification, facilitating the expression of recessive traits (Ronfort and Glemin 2013). This process is critical for advancing germplasm research, developing new germplasm, and optimising the use of heterosis, as exemplified by its prominent role in the development and application of maize inbred lines (Li et al. 2022).

While inbreeding depression is a common consequence of selfing, its severity varies markedly across species, populations, and individuals (Collin et al. 2009). In some cases, selfed progeny perform as well as or better than outcrossed ones, as observed in *Mimulus guttatus* (Murren and Dudash 2012) and *Ceiba pentandra* (Lobo et al. 2015). Several conifers, including *Pinus radiata* , *P. resinosa* , *Picea omorika* , and *Thuja plicata* , exhibit low inbreeding depression (El‐Kassaby et al. 1994; Wu et al. 2016). Notably, some selfed *P. radiata* families showed superior stem growth (Wu et al. 1998), and increased selfing rates even enhanced offspring performance in *P. taeda* (Ford et al. 2015). Despite a high selfing rate, *Foetidia mauritiana* maintained substantial genetic diversity (Cuénin et al. 2019). These findings suggest that certain genotypes may possess mechanisms that buffer or bypass the detrimental effects typically associated with inbreeding.

Indeed, inbreeding depression often affects multiple plant life stages, especially seed development, germination, and seedling establishment (Kephart et al. 1999; Naito et al. 2005; Collin et al. 2009). Selfing commonly reduces seed quality, germination, and survival (Darwin 1877; Collin et al. 2009; Rymer et al. 2015; Dieterich Mabin et al. 2021), though some species or genotypes show minimal depression in early‐stage vigour (Nason and Ellstrand 1995; Speroni et al. 2014). Consistent with reports of limited early‐stage inbreeding depression in some species, our recent findings revealed high selfed seed vigour in a *Cunninghamia lanceolata* parent line (Deng et al. 2022, 2024). So far, studies of this phenomenon have primarily focused on phenotypic traits, while the underlying physiological and genetic mechanisms remain largely unexplored.

Seed vigour, a composite trait encompassing seed longevity, germination speed, radicle and hypocotyl growth, and early stress tolerance, is critical for successful seedling establishment and is heavily influenced by both genetic and environmental factors (Ali et al. 2021; Reed et al. 2022). Among these factors, genetics play a predominant role, with mating systems (e.g., outcrossing, inbreeding, and selfing) significantly affecting seed vigour by influencing traits such as seed quality, germination, and early growth (Kalisz 1989; Gargano et al. 2011; Saux et al. 2020). The regulation of seed vigour involves diverse biological processes, including phytohormone signalling, oxidative stress response, enzyme activity, transcription factors (such as MYB family), DNA and RNA repair, cell wall modification (response of cell wall loosening genes, such as *EXP*), nucleoside, nucleotide, sugar and energy metabolism, as well as environmental factors (Rajjou et al. 2012; Ventura et al. 2012; Domergue et al. 2019; Li et al. 2021; Niehaus et al. 2022; Reed et al. 2022; Waterworth et al. 2022; Huang et al. 2024; Lv et al. 2024). Seed germination, a key determinant of vigour, requires efficient nucleotide metabolism to support nucleotide biosynthesis and coordinated expression of genes (Price and Murray 1969). Nucleotide analysis could serve as a useful method for the rapid assessment of seed lot viability or vigour (Standard et al. 1983). Moreover, genes associated with nucleoside metabolism are transcriptionally upregulated, with adenine salvage pathways (purine metabolism) playing a pivotal role in sustaining energy demands and growth (Lee and Moffatt 1994; Bray 2017). Despite its importance, the molecular basis of purine metabolism's role in seed vigour remains largely unexplored, particularly in selfed seeds.

Unfortunately, due to elevated homozygosity and expression of deleterious recessive alleles, selfing complicates the study of seed vigour by triggering inbreeding stress and disrupting gene regulation, which obscures the underlying physiological and genetic mechanisms. While direct evidence on gene regulation of seed vigour in selfed progeny remains scarce, studies on selfing‐induced transcriptional changes have provided a molecular basis that helps guide the exploration of underlying mechanisms. Evidence from plant and animal systems shows that inbreeding can activate compensatory mechanisms, such as upregulation of molecular chaperones (e.g., HSP70) and vacuolar ATPase subunits, to mitigate protein misfolding and maintain cellular homeostasis (Kristensen et al. 2002; Pedersen et al. 2005; Cheng et al. 2006; Bahrndorff et al. 2010; Menzel et al. 2015). These responses are often accompanied by increased expression of genes involved in nucleotide, sugar, and energy metabolism, indicating metabolic adjustments are vital under inbreeding stress. Inbred lines also tend to enhance expression of genes linked to ATP biosynthesis, proteasome activity, and compatible solutes like maltose, reflecting a shift toward maintaining cellular function (Kristensen et al. 2005; Pedersen et al. 2008). However, whether stress‐responsive genes are consistently up‐ or downregulated in selfed progeny remains controversial (Kariyat et al. 2012; Menzel et al. 2015), suggesting a complex and context‐dependent regulatory landscape. It is still unclear whether these transcriptional responses directly influence seed vigour traits such as germination and stress tolerance. This uncertainty underscores a key knowledge gap: the mechanistic link between inbreeding‐responsive gene expression and seed vigour remains unresolved.

*Cunninghamia lanceolata* , a fast‐growing evergreen timber species in the Cupressaceae family, is widely cultivated in southern China for its economic value (Yang et al. 2022). Known for its outcrossing nature, *C. lanceolata* typically exhibits elevated inbreeding depression in its selfed progeny, leading to reduced vigour and, in some cases, mortality (Huang et al. 2022). However, the identification of a self‐fertilising parent with low inbreeding depression provides a unique opportunity to explore the mechanisms that sustain seed vigour under selfing conditions (Deng et al. 2022). This study integrates pollination experiments (selfing, inbreeding, and non‐inbreeding) with phenotypic evaluations, as well as transcriptomic analysis, to uncover the genetic and molecular mechanisms underlying seed vigour in inbred progenies of *C. lanceolata* . Our findings highlight the critical role of purine metabolism in seed vigour and provide new insights into how seed vigour is maintained during inbreeding, while also offering new germplasm resources for the development of higher‐quality seed orchards in *Cunninghamia lanceolata* .

## 2 Materials and Methods

### 2.1 Plant Materials

The Chinese fir ( *C. lanceolata* ) clone ‘cx569’ has been identified as a self‐fertilising parent (Deng et al. 2022). Seeds were produced by self‐pollination, inbred (backcrossed) pollination, and non‐inbred pollination of this clone in a 2.5‐generation seed orchard located at the Xiaokeng State Forest Farm (Shaoguan, Guangdong Province, China; 24°70′N, 113°81′E, altitude of 328–339 m). For inbred pollination, clone cx569 served as the pollen receptor, while its first filial generation parents (cx837, cx840, cx851, cx860, cx865, cx870, cx871 and cx872), along with parent cx569 itself, were selected as pollen donors, based on the pedigree relationships of the Chinese fir parent trees in the orchard (Figure S1A) (Zeng et al. 2023). Additionally, a positive control (non‐inbreeding) was included, comprising a mixture of four unrelated parents (cx400, cx844, cx850 and cx969) used as non‐inbred pollen donors mixed in equal proportions (Figure S1B). The different pollination combinations included cx569 × cx837 (denoting clone cx569 ♀ as maternal parent and cx837 ♂ as the paternal parent) cx569 × cx837, cx569 × cx840, cx569 × cx851, cx569 × cx860, cx569 × cx865, cx569 × cx870, cx569 × cx871, cx569 × cx872 and selfing (cx569 × cx569). By convention, the female parent is listed first in these cross designations. All pollination trials were conducted in early March 2021. Fresh seeds were collected from pollinated female cones in early October 2021 and subsequently stored at 4°C for germination experiments.

### 2.2 Evaluation of Seed Quality and Seed Vigour

Seed quality indicators, including cone‐seed rate, seed soundness, thousand kernel weight, astringent seed rate, empty seed rate, embryo abortion rate, and seed vigour indicators, including seed germination rate, germination potential, radicle length and hypocotyl length, were calculated. Prior to germination, fresh seeds were sterilised by immersion in a 0.2% potassium permanganate aqueous solution for 20 min, followed by three rinses with distilled water. To break seed dormancy, the sterilised seeds were placed in distilled water for 24 h in an artificial climate incubator maintained at 25°C under dark conditions. For germination, the seeds were placed on moist filter paper in 9.0 cm diameter Petri dishes and incubated in an artificial environment at 25°C with a 16‐h light/8‐h dark cycle at the Guangdong Academy of Forestry. Seeds were classified as germinated when the radicle extended 1 mm beyond the seed coat. Seedlings were considered established when the cotyledons began to open and the first true leaves reached the pre‐emergence stage. The germination potential percentage was calculated after 7 days of germination. The germination rate was recorded daily and the experiment concluded after 14 days. Additionally, root and hypocotyl lengths of germinated seeds were measured.

### 2.3 Vibratome Cutting

Radicles and hypocotyls from 14‐day‐old seedlings were prepared for histological sectioning. The sampling sites of the radicle and the hypocotyl were taken from the middle region of the radicle and the hypocotyl, respectively. Fresh radicles and hypocotyls were embedded in 5% (w/v) low‐melting‐point analytical agarose (Promega). The agarose blocks containing the samples were cut into cubes and affixed to the blade of a Vibratome VT 1200S (Leica Microsystems, Wetzlar, Germany). Longitudinal sections, 40 μm thick, were sliced and mounted on slides with water. The sections were immediately stained with 0.1% (w/v) Toluidine blue O (TBO, Sigma) for 60 s and observed under an Olympus CX31 microscope equipped with a computer‐assisted digital camera (Olympus Corp., Tokyo, Japan).

### 2.4 Radicle and Hypocotyl Sampling of Inbred Seed and mRNA‐Seq Library Preparation

After 14 days of germination (prior to the first true leaves stage), radicles and hypocotyls were promptly isolated from germinated seeds, with 200 mg of tissue collected from each sample. The samples were immediately snap‐frozen in liquid nitrogen for subsequent RNA extraction. Three biological replicates were prepared for each inbred seed germination treatment. Total RNA was extracted using the RNeasy PowerPlant Kit (13500‐50; Qiagen), and genomic DNA was removed using the Turbo DNA‐free Kit (AM1907; Invitrogen). The quantity and purity of the RNA samples were assessed with a NanoPhotometer spectrophotometer (Implen, Westlake Village, CA, USA), while RNA concentration was measured using the Qubit RNA Assay kit and Qubit 2.0 Fluorometer (Life Technologies, Carlsbad, CA, USA). RNA integrity was evaluated using the RNA Nano 6000 Assay kit and the Bioanalyzer 2100 system (Agilent Technologies, Santa Clara, CA, USA). cDNA libraries were prepared from 1000 ng of RNA using the KAPA mRNA Hyper Prep Kit (KK8581; Roche) with the KAPA Dual‐indexed Adapter Kit for Illumina platforms (KK8722; Roche). The libraries were sequenced on an Illumina HiSeq X Ten platform by the Guangzhou Science Corporation of Gene (http://www.scgene.com/), generating paired‐end reads.

### 2.5 Bioinformatic Analysis

After filtering adapter sequences and low‐quality reads from the raw data, the clean reads were aligned to the *C. lanceolata* genome using HISAT v 2.04 software with default parameters (Kim et al. 2015). The *C. lanceolata* genome data was obtained from the whole‐genome shotgun sequencing project (GenBank: BSBN00000000.1). Transcript expression levels were normalised using fragments per kilobase of transcript sequence per million base pairs sequenced (FPKM). Differentially expressed genes (DEGs) between selfed seeds and inbred/non‐inbred seeds were identified using the DESeq2 R package (version 1.10.1), with a cut‐off *p* < 0.05 and |log_2_ FC (fold change, FC)| > 1 (Love et al. 2014). Gene Ontology (GO) enrichment analysis was carried out using the TBtools software under default parameters (Chen et al. 2023). GO network analyses were conducted with the ClueGO plug‐in of Cytoscape software, using the *Arabidopsis thaliana* database as the reference background, and default settings for all other parameters.

### 2.6 cDNA Synthesis and Quantitative Real‐Time PCR (qRT‐PCR)

Total RNA was extracted from embryos, radicles, and hypocotyls, and by reverse transcription using a kit (Vazyme Biotech Co. Ltd). Quantitative reverse transcription polymerase chain reaction (qRT‐PCR) was performed using the ChamQ Universal SYBR qPCR Master Mix (Vazyme, Nanjing, China) as described previously (Yang et al. 2023). Transcript quantification was conducted using a Bio‐Rad CFX96 system with a kit from Takara Biosystems. The relative expression levels of messenger RNA (mRNA) were calculated using the 2^−ΔΔCt^ method (Livak and Schmittgen 2001), with *GAPDH* serving as the reference gene (Bao et al. 2016). The primers used for qRT‐PCR are listed in Table S1.

### 2.7 Enzyme Activity Analysis

The adenine phosphoribosyl transferase (APRT) enzyme activities of embryos, radicles, and hypocotyls, each sampled at equal fresh weight, were measured using a Plant APRT Enzyme‐Linked Immunosorbent Assay (ELISA) Kit (Shenzhen Ziker Biological Technology Co. Ltd. (www.zikerbio.com)) following the manufacturer's instructions. Optical density (O.D.) values at 450 nm were recorded within 15 min using an enzyme‐labelled instrument. The APRT enzyme activities were calculated based on the O.D. values, as specified by the manufacturer's instructions.

### 2.8 Transformation Vector, Construction of Transgenic Plant and Growth Conditions

To generate an overexpression line, the CDS of the *ClAPRT3* gene was amplified by PCR from the cDNA of the parent cx569. A transgenic *Arabidopsis thaliana* line overexpressing *ClAPRT3* was created by cloning the *ClAPRT3* gene into the *PBI121‐eGFP* vector under the control of the 35S promoter. The recombinant plasmid was transformed into Agrobacterium strain GV3101 and then introduced into *A. thaliana* (Col‐0) using the floral dip method. A homozygous line was selected on MS medium containing 50 mg/L kanamycin, with three lines selected for each overexpression construct.

Surface‐sterilised seeds were plated at MS medium and stratified for 2 days at 4°C. Then the plates were transferred to the greenhouse at 23°C under a 16‐h light/8‐h dark photoperiod.

### 2.9 Seed Treated With APRT Inhibitor and Substrate for Germination

For the concentration screening experiment on adenine phosphoribosyl transferase (APRT) enzyme inhibitors, 6‐diaminopurine (DAP) was applied to open‐pollinated seeds at concentrations of 0, 10, 25, 50, 100, 500, 1000 and 5000 μmol·L^−1^, based on concentration settings from a previous study (Victor et al. 1999). Similarly, the concentration screening of APRT substrate adenine was applied at concentrations of 0, 10, 20, 50, 100, 500, 1000, 5000 and 10 000 μmol·L^−1^. Before germination, inbred and non‐inbred seeds were soaked in an aqueous solution containing DAP or adenine and treated in darkness at 20°C for 24 h. The seeds were placed on moist filter paper in Petri dishes and incubated under controlled conditions at 25°C with a 16‐h light/8‐h dark cycle. Daily watering was performed using water containing the respective concentration of DAP or adenine. For AMP rescue assays, adenine or AMP was added with a final concentration range between 1 and 1.5 mM. The control group was treated with sterile ddH_2_O.

### 2.10 Analyses of Purine Metabolites by LC–MRM–MS

Absolute quantitation of adenine (ADE), adenosine monophosphate (AMP), adenosine diphosphate (ADP), and adenosine triphosphate (ATP) was performed using Liquid Chromatography‐Mass Spectrometry (LC–MS), following the protocol provided by Agilent Technologies (Santa Clara, California, United States). Radicle (100 mg) or hypocotyls (100 mg) were harvested, washed with sterile water, and thoroughly dried with paper towels. Samples were immediately snap‐frozen in liquid nitrogen, ground into a fine powder, and transferred to 2‐mL safe‐lock centrifuge tubes. Then exact sample weights were recorded for concentration calculations. Each sample was extracted with 2 mL 50% acetonitrile solution, vortexed thoroughly, and centrifuged at 8000 × *g* for 10 min at 4°C. The supernatant was collected and transferred to autosampler vials for LC–MS analysis. Chromatographic separation was performed using an Agilent 1260 Infinity system (Santa Clara, California, United States) coupled with an AB SCIEX QTRAP 5500 (Fremont, California, United States) mass spectrometer. Separation utilised an Agilent Poroshell 120 HILIC‐Z column (2.7 μm) under the following conditions: mobile phases, solvent A, 100 mM ammonium acetate (pH 8.9), solvent B, 100% acetonitrile (CAN). The elution gradient (v/v) was 50% A and 50% B at 0 min, 90% A and 10% B at 10 min, 50% A and 50% B at 10.1 min, 50% A and 50% B at 20 min. The column flow rate was 0.25 mL/min; the column temperature was 30°C; the injection volume was 5 μL. The QTRAP‐MS was operated in Multiple Reaction Monitoring (MRM) mode with positive ion polarity using the following settings: ion source voltage, 4500 V; drying N_2_ gas and temperature, 40 L/min and 600°C respectively; collisionally activated dissociation, medium; entrance potential, 10 V; and nebulizer gas pressure, 50 psi. Optimised MRM transitions for each target metabolite were listed in Table S2. Data analysis: quantification of metabolites was carried out using AB SCIEX Analyst Software (version 1.6.1, Build 3773). The integrated peak areas were compared with calibration curves generated from external standards. Calibration curves were deemed valid if the *R* ^2^ value exceeded 0.99 and the corresponding *p‐*value was < 0.001. Metabolite concentrations were normalised to the initial fresh weight of the samples.

### 2.11 Yeast One‐Hybrid Assay

The full‐length coding sequence (CDS) of the candidate *ClMYB32* in Chinese fir was cloned into the pGADT7 vector, while the promoter fragment of *ClAPRT3* was cloned into the pAbAi vector. The constructed pAbAi plasmid was linearized using the restriction enzyme BstBI and subsequently transformed into Y1HGold yeast strain. The yeast one‐hybrid (Y1H) assays were conducted as described previously (Yang et al. 2020). Transformants were plated on solid SD/−Leu medium containing 800 ng/mL of aureobasidin A (AbA) for assessment. An empty pGADT7 vector served as the negative control. The primers utilised for plasmid construction are detailed in Table S3.

### 2.12 Dual‐Luciferase Assay

The promoter of *ClAPRT3* was cloned into the pGreen II 0800‐LUC vector, while the full‐length CDSs of *ClMYB32* were cloned into the pGreen II 0029 62‐SK vector. Dual‐luciferase assays were performed via transient expression in tobacco leaves, following Yang et al. (2020). The primers used for plasmid construction were listed in Table S3.

### 2.13 Promotor and Coding Sequence Cloning and Cis‐Element Analysis

Promoter fragments were amplified using DNA templates of cx569 × cx837, cx569 × cx569, cx569 × cx840 and non‐inbred progenies, and the parent cx569. For the coding sequence, RNA extracted from cx569 × cx837, cx569 × cx569, cx569 × cx840, non‐inbred progenies, and the parent cx569 was used as the template. The amplified products were purified, ligated into the pEASY‐Blunt vector, and subsequently introduced into *E. coli* . Positive clones were identified by PCR screening and verified by sequencing.

### 2.14 Molecular Docking

Molecular docking experiments were implemented to explore the interaction between the receptor adenine phosphoribosyl transferase 3 (CIAPRT3) and its substrate ligand, adenine. The three‐dimensional structural data of adenine was retrieved from the PubChem database (https://pubchem.ncbi.nlm.nih.gov/). The protein structure for CIAPRT3 was obtained from the AlphaFold3 server (https://alphafoldserver.com/). Docking simulations were performed using AutoDock Vina (version 1.5.7) (https://ccsb.scripps.edu/mgltools/downloads/) and PyMOL software (version 2.6.0). The resulting three‐dimensional docking results were further converted into two‐dimensional representations using Discovery Studio software (version 4.5).

### 2.15 Data Analysis

Statistical analysis, including analysis of variance (ANOVA) and principal component analysis (PCA), was performed using IBM SPSS Statistics 26.0 (IBM Corp., Armonk, NY, USA). Student's *t*‐test and one‐way ANOVA followed by Duncan's multiple comparison test were used to assess variance among mean values. Statistical significance was indicated by asterisks: *p < 0.05; **p < 0.01; ***p < 0.001; ****p < 0.0001.

## 3 Results

### 3.1 Selfed Seed Quality and Vigour Evaluation

**FIGURE 1:**
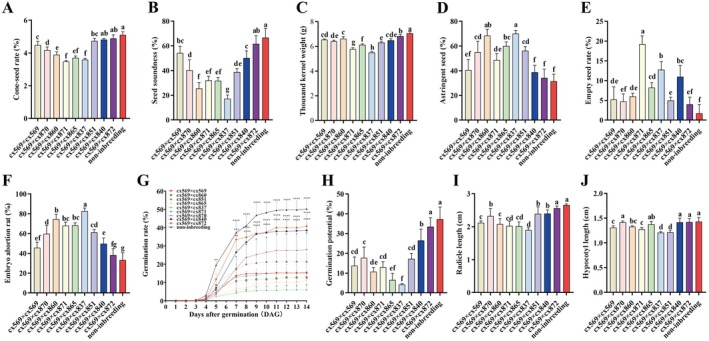
Assessment of inbred seed quality and vigour. (A) The cone‐seed rate, (B) seed soundness, (C) thousand kernel weight, astringent seed rate (D), empty seed rate (E), embryo abortion rate (F), seed germination (G), germination potential in 7 days after germination (H), radicle (I) and hypocotyl length (J) (14 days after germination). Each experiment was repeated six times with 100 seeds in each replication. Values represent the mean ± SD. Different letters denote statistically significant differences resulting from Duncan's test following one‐way ANOVA. *p < 0.05, **p < 0.01, ***p < 0.001, ****p < 0.0001 (Student's *t*‐test).

Extensive variation was observed among selfed, inbred, and non‐inbred seeds across six quality indicators (cone‐seed rate, seed soundness, thousand kernel weight, astringent seed rate, empty seed rate, and embryo abortion rate) and four vigour‐related traits, including seed germination rate, germination potential, radicle length, and hypocotyl length. The cone‐seed rate ranged from 3.5% to 5.1%, with cx569 × cx871, cx569 × cx865, and cx569 × cx837 producing the fewest seeds, while the non‐inbred group yielded the most (Figure 1A). Interestingly, cx569 × cx569 (selfing) produced an intermediate 4.5% seeds per cone, but significantly lower than cx569 × cx840, cx569 × cx872, and the non‐inbred group. The seed soundness of the selfing group (54.3%) ranked third among all groups, three times higher than that of cx569 × cx837 (17.3%) but 12.5% lower than the non‐inbreeding group (66.8%) (Figure 1B). For the thousand kernel weight index, selfed seeds were significantly heavier than seeds from some inbred groups, such as cx569 × cx871, cx569 × cx865, and cx569 × cx837 (Figure 1C). Regarding embryonic lethal seeds, the astringent seed rate of the selfing group (40.5%) was significantly lower than that of most inbred groups, such as cx569 × cx870, cx569 × cx860, cx569 × cx865, cx569 × cx837 and cx569 × cx851, which ranged from 55.0% to 70.0% (Figure 1D). However, no significant difference was observed between the selfing group and cx569 × cx871 (48.8%), cx569 × cx840 (38.8%), cx569 × cx872 (34.3%), or the non‐inbreeding group (31.5%). Additionally, the empty seed rate in the selfing group (5.3%) was significantly lower than that of cx569 × cx871 (19.3%), cx569 × cx837 (12.8%) and cx569 × cx840 (11.0%) (Figure 1E). For embryo abortion, the selfing group (45.8%) exhibited a significantly lower embryo abortion rate compared to most inbred groups, such as cx569 × cx870 (59.8%), cx569 × cx860 (74.5%), cx569 × cx871 (68.0%), cx569 × cx865 (68.3%), cx569 × cx837 (82.8%) and cx569 × cx851 (61.3%) (Figure 1F). However, the selfing group still had a significantly higher embryo abortion rate than the non‐inbred group (33.3%). These results suggest that selfed seeds exhibit relatively high seed quality compared to certain inbred groups and do not result in the highest rate of embryonic lethal seeds.

To further assess seed quality and vigour among selfing, inbred, and non‐inbred groups, seed germination experiments were conducted. Dynamic germination analysis revealed that selfed seeds did not consistently exhibit the lowest germination rates. From Day 7 onward, selfed seeds showed a significantly higher germination rate compared to the inbred group (cx569 × cx837), though it remained lower than that of several other inbred groups (cx569 × cx870, cx569 × cx840 and cx569 × cx872) and the non‐inbred group (Figure 1G). By Day 14, the germination rate for selfed seeds reached 15.3%, substantially higher than the 6.0% observed in cx569 × cx837. However, this rate was still lower than that of cx569 × cx870 (28.0%), cx569 × cx840 (38.5%), cx569 × cx872 (40.8%), and the non‐inbred group (50.3%). The non‐inbred seeds exhibited the highest germination potential (37.3%), while cx569 × cx837 seeds showed the lowest at 4.3% (Figure 1H). The germination potential of the selfed seeds reached 13.8%, which was twice as high as that of cx569 × cx865 (6.5%) and three times higher than that of cx569 × cx837 (4.3%). While several inbred seeds displayed low germination potential (< 20%), some, such as cx569 × cx840 and cx569 × cx872, exhibited relatively high germination potentials of 26.5% and 33.5%, respectively. Moreover, root length (Figure 1I) and hypocotyl length (Figure 1J) on Day 14 after germination for self‐pollinated seeds were not the lowest compared to certain inbred seeds, such as cx569 × cx837 and cx569 × cx851. These results indicate that while selfing may not produce the most vigorous seeds overall, it consistently outperformed some inbred groups and maintained moderate germination performance and seedling growth metrics.

Principal component analysis (PCA) was conducted to evaluate 10 vigour indicators. Prior to the analysis, the Kaiser‐Meyer‐Olkin (KMO) test indicated a measure of sampling adequacy of 0.72, and the Bartlett's test of sphericity yielded a *p* value of less than 0.001 (Table S4), confirming the dataset's suitability for PCA. Components with eigenvalues greater than 1 were extracted, resulting in two principal components that accounted for 76.26% and 10.26% of the variance, respectively, with a cumulative contribution of 86.52% (Table S5). Comprehensive evaluation indicators were analysed, and score coefficients for each indicator were calculated based on the main factor eigenvalues and component matrix (Table S6). Using the comprehensive score (CS) derived from seed vigour evaluation, the seeds were classified into four major groups: Grade I (extreme‐high‐vigour): non‐inbred seeds (CS > 4.00); Grade II (high‐vigour): cx569 × cx872 and cx569 × cx840 (1.00 ≤ CS < 4.00); Grade III (medium‐vigour): cx569 × cx870, selfing, cx569 × cx851 and cx569 × cx860 (−1.00 ≤ CS < 1.00); Grade IV (low‐vigour): cx569 × cx865, cx569 × cx871 and cx569 × cx837 (−4.00 ≤ CS < −1.00) (Table S7). These results highlight significant variability in seed quality and vigour among the groups. Non‐inbred seeds exhibited the highest vigour, followed by the high‐vigour inbred seeds (cx569 × cx840 and cx569 × cx872). In contrast, cx569 × cx837 consistently displayed the lowest seed vigour. Selfed seeds were classified within the medium‐vigour group, demonstrating intermediate performance relative to inbred and non‐inbred groups. These findings provide a robust framework for understanding the variability in selfed seed vigour and identifying potential factors influencing seedling establishment and growth. Further investigation into key vigour indicators, such as radicle and hypocotyl lengths, is warranted, as these traits are critical for successful transition from seed to seedling.

### 3.2 Radicle and Hypocotyl Elongation Distinguish Inbred Seeds of Varying Vigour in Germination Assays

**FIGURE 2:**
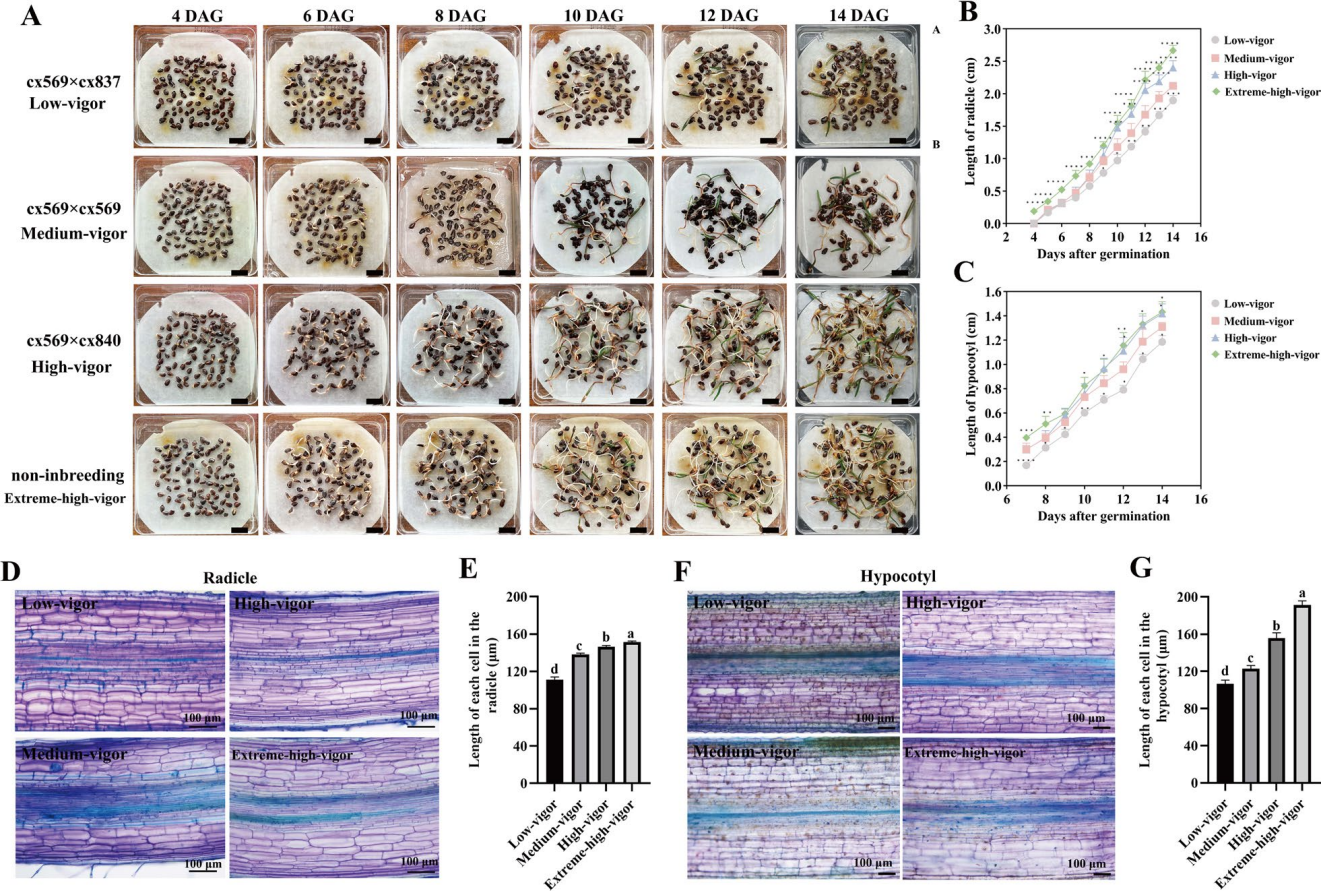
Dynamic germination of low‐vigour (cx569 × cx837), medium‐vigour (cx569 × cx569), high‐vigour (cx569 × cx840), and extreme‐high‐vigour (non‐inbred) seeds. (A) Dynamic germination of inbred seeds at 4 days after germination (DAG), 6 DAG, 8 DAG, 10 DAG, 12 DAG, and 14 DAG. The black scale represents 1.0 cm. (B, C) Dynamic growth elongation of radicle and hypocotyl during inbred germination. Each experiment was repeated six times with 100 seeds in each replication. Values represent the mean ± SD. *p < 0.05, **p < 0.01, ***p < 0.001, ****p < 0.0001 (Student's *t*‐test). Longitudinal sections of radicles of low‐vigour, medium‐vigour, high‐vigour and extreme‐high‐vigour seeds at 14 DAG (D). The length (E) of each cell were measured in the radicles at 14 DAG. Longitudinal sections of hypocotyls of low‐vigour, medium‐vigour, high‐vigour, and extreme‐high‐vigour seeds (F) seeds at 14 DAG. The length (G) of each cell were measured in the hypocotyls at 14 DAG. Error bars indicate a standard deviation of six biological replicates. Each biological replicate consists of 5 seeds, with the cell lengths in the radicle and hypocotyl measured separately. Different letters denote statistically significant differences resulting from Duncan's test following one‐way ANOVA.

Radicle and hypocotyl lengths are critical for the successful transition from seed to seedling and are widely recognised as key traits reflecting seed vigour. Based on the previous results, we further analysed the dynamic elongation of the radicle and hypocotyl during seed germination in selfed seeds. We randomly selected representative groups for dynamic seed germination observations, focusing on radicle and hypocotyl elongation. The selected groups included the extreme‐high‐vigour group (non‐inbred), the high‐vigour group (cx569 × cx840), the medium‐vigour group (selfing), and the low‐vigour group (cx569 × cx837). By Day 4 of germination, medium‐vigour seeds demonstrated a higher percentage of seeds breaking through the seed coat (10.6%) compared to the low‐vigour seeds (5.4%) (Figure 2A). However, both low‐vigour and medium‐vigour seeds exhibited significantly fewer seeds breakthroughs compared to high‐vigour seeds (27.4%) and extreme‐high‐vigour seeds (31.2%). Initially, the radicle lengths of medium‐vigour seeds were not significantly different from those of low‐vigour seeds, but both were notably shorter than the radicle lengths of extreme‐high‐vigour seeds (Figure 2B). This pattern persisted until Day 8 of germination. However, by Day 9, a shift in growth pattern was observed. On Day 9 of germination, the mean radicle length of medium‐vigour seeds (mean = 1.0 cm) was significantly longer than that of low‐vigour seeds (mean = 0.8 cm), but shorter than that of extreme‐high‐vigour seeds (mean = 1.3 cm). By Day 10 onward, the radicle length of medium‐vigour seeds remained significantly shorter than that of both high‐vigour seeds and extreme‐high‐vigour seeds, but consistently exceeded that of low‐vigour seeds. This trend continued until Day 14 (end of seed germination), when cotyledons were fully expanded, and true leaves began to emerge. For hypocotyl elongation, medium‐vigour seeds consistently outperformed low‐vigour seeds from Day 7 through Day 14 (Figure 2C). On Day 14, the hypocotyl lengths of medium‐vigour and low‐vigour seeds reached 1.2 and 1.3 cm, respectively; both measurements were significantly shorter than those of high‐vigour and extreme‐high‐vigour seeds, which each reached 1.4 cm.

Histological analysis of radicle sections on the 14th after germination revealed significant differences in cell length among seeds from various groups (Figure 2D). Extreme‐high‐vigour seeds exhibited the longest cells (mean = 151.30 μm), followed by high‐vigour seeds (mean = 146.19 μm), medium‐vigour seeds (mean = 138.27 μm), and low‐vigour seeds, which had the shortest radicle cells (mean = 111.05 μm) (Figure 2E). A similar trend was also observed in the hypocotyl cells (Figure 2F). The cell lengths in the hypocotyls of medium‐vigour seeds (mean = 122.8 μm) were significantly longer than those of low‐vigour seeds (mean = 106.5 μm). However, both medium‐ and low‐vigour seeds exhibited considerably shorter hypocotyl cells compared to high‐vigour (mean = 155.6 μm) and extreme‐high‐vigour seeds (mean = 191.3 μm) (Figure 2G). These findings from the dynamic germination assays suggest that selfing results in a moderate level of seed vigour, particularly in radicle and hypocotyl development. The phenotypic differences in radicle and hypocotyl elongation between selfed and non‐inbred seeds highlight the potential of selfed seeds to maintain an intermediate level of vigour. Furthermore, these results provide phenotypic evidence for exploring potential mechanisms regulating the vigour of selfed seeds. Such mechanisms warrant further investigation to understand their roles in seed development and performance.

### 3.3 Transcriptomic Analysis Highlights Purine Metabolism as a Key Process in Selfed Seed Vigour

To gain deeper insights into the mechanism underlying the maintenance of selfed seed vigour, RNA sequencing (RNA‐Seq) was performed on radicles and hypocotyls from low (cx569 × cx837), medium (cx569 × cx569), high (cx569 × cx840), and extreme‐high‐vigour (non‐inbred) seeds on Day 14 of germination. Samples were collected in triplicates for group analysis. The analysis yielded high‐quality data, with more than 93% of clean reads successfully aligned to the *C. lanceolata* reference genome (Table S8). To elucidate the transcriptome dynamics among the seed group, correlation analyses were performed, revealing strong positive correlations between replicates within each sample (*R* ^2^ > 0.90) (Table S9). A Principal component analysis (PCA) further demonstrated a distinct transcriptomic landscape in both radicle (Figure S2A) and hypocotyl (Figure S2B) of the medium‐vigour seed, which were clearly separated from the low, high, and extreme‐high‐vigour groups. These findings highlight transcriptional differences among seeds of varying vigour levels, indicating that selfed seeds exhibit unique gene expression profiles compared to other inbred and non‐inbred seeds.

To further identify gene sets associated with seed vigour of selfed progeny, differential gene expression (DGE) analysis was performed for the three comparisons: medium‐vigour versus low‐vigour, high‐vigour versus medium‐vigour and extreme‐high‐vigour versus medium‐vigour. The analysis revealed 2904 and 3123 differentially expressed transcripts in the radicle and hypocotyl, respectively, with a similar number of DEGs identified in both tissues (Figure S3A). Moreover, there were different numbers of DEGs across differential comparison groups in the radicle and hypocotyl tissues (Figure S3B). Whether in the radicle or hypocotyl, in the comparison group medium‐vigour versus low‐vigour, the number of upregulated differential genes was lower than that of downregulated genes. In contrast, in high‐vigour versus medium‐vigour, the trend was reversed, with upregulated differential genes outnumbering downregulated ones. This result indicates that there are significant differences in the number of differentially expressed genes between the comparison groups with varying vigour. To explore the biological pathways primarily involved in the upregulated and downregulated genes, we conducted GO enrichment analysis. The results revealed that, both in the radicle and hypocotyl, upregulated genes were enriched not only in stress‐related pathways but also predominantly in pathways associated with growth and development, such as cell wall biosynthesis, xyloglucan metabolism, energy metabolism and nucleotide metabolism (Figure S3C,D). In contrast, downregulated differential genes were mostly enriched in pathways related to basic metabolism and oxidative stress (Figure S3E,F). These findings further suggest that the gene expression of relatively high‐vigour seeds tends to promote growth and development, while the relatively low‐vigour group is more focused on defence responses. Notably, the enrichment patterns in the radicle and hypocotyl showed similarity.

**FIGURE 3:**
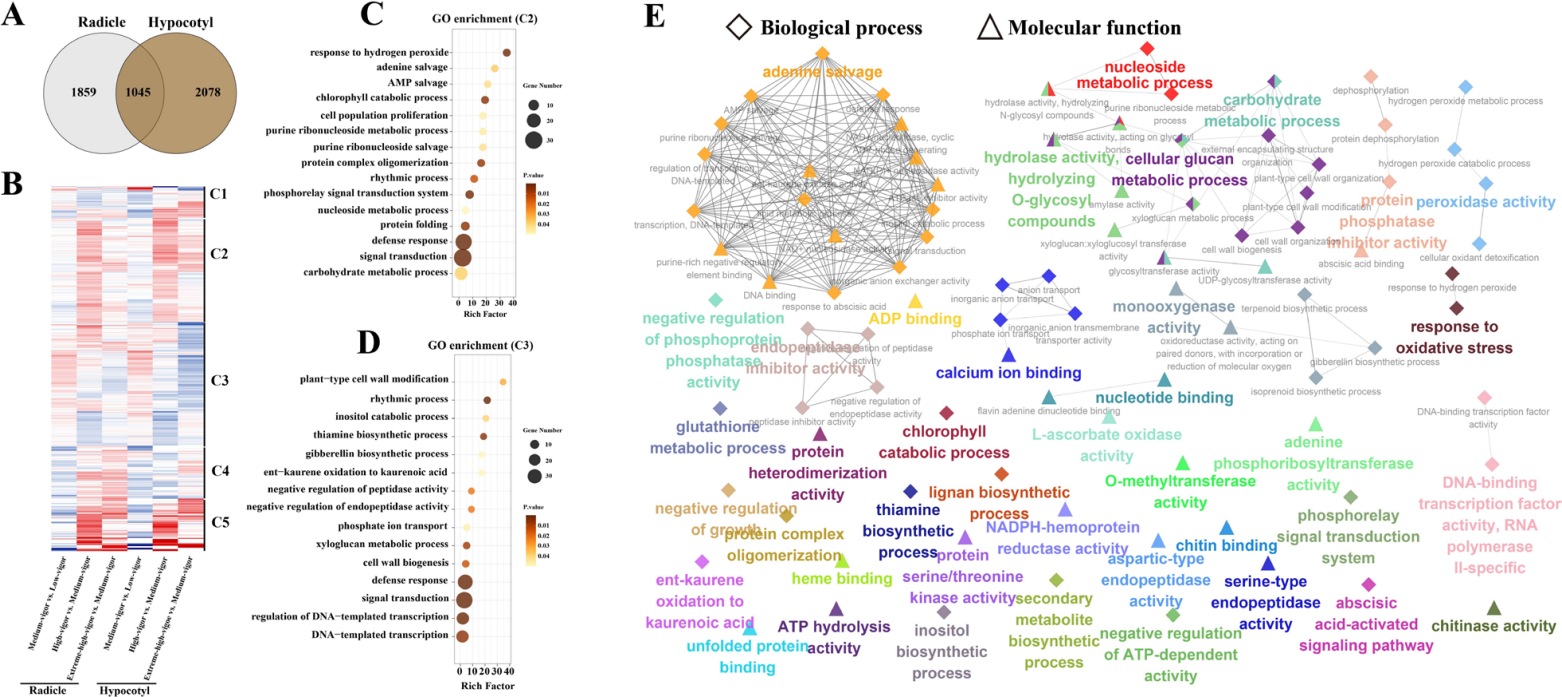
Analyses of differentially expressed genes (DEGs) of overlapped DEGs between radicle and hypocotyl. (A) Venn analysis of DEGs between radicle and hypocotyl. (B) Heatmap analysis of 1045 DEGs. The character C1 on the right side of the heatmap denotes cluster 1. Similarly, C2, C3, C4 and C5 indicate cluster 2, cluster 3, cluster 4, and cluster 5. Graph (C) and (D) indicate the results of GO enrichment analysis of biological process from C2, and C3, respectively. (E) Network of the biological processes and molecular function from detected 1045 DEGS using the ClueGO plu‐in of the Cytoscape software.

To further explore the similarity between the radicle and hypocotyl, we performed a Venn diagram analysis of the differentially expressed genes (DEGs) in both tissues. The Venn analysis revealed that 36.0% (1045) of the radicle DEGs overlapped with 33.5% of the hypocotyl DEGs (Figure 3A), with similar numbers of unique DEGs in each tissue. Additionally, heatmap clustering of the overlapping DEGs (1045) divided them into five distinct clusters (C1, C2, C3, C4 and C5), each showing similar expression patterns in both the radicle and hypocotyl across the comparison groups (Figure 3B). For instance, clusters C2 and C4 exhibited upregulated expression patterns across all comparisons (medium‐vigour vs. low‐vigour, high‐vigour vs. medium‐vigour, and extreme‐high‐vigour vs. medium‐vigour). Clusters C3 showed an upregulated‐downregulated‐downregulated expression profile, while clusters C5 displayed a trend of downregulation‐upregulation‐upregulation. Despite these variations, the expression patterns of these clusters were remarkably consistent between the radicle and hypocotyl, underscoring the presence of similar regulatory mechanisms in both tissues during selfing.

To identify biological pathways associated with selfed seeds vigour, we performed Gene Ontology (GO) enrichment analysis on 1045 overlapped DEGs (Table S10). In cluster C1, we observed significant enrichment of biological processes related to cell wall dynamics, including xyloglucan metabolism, cell wall biogenesis, and cell wall organisation (Figure S4A) and hydrolase activity was also highly enriched in molecular function (Figure S4D). Cluster C2 was characterised by enriched processes involved in nucleotide metabolism, such as adenine and AMP salvage, purine ribonucleoside metabolism and nucleoside metabolism, along with growth‐related processes such as cell population proliferation and the rhythmic process (Figure 3C). Correspondingly, nucleoside‐related enzyme activity and binding were prominent molecular functions in C2 (Figure S4E). Cluster C3 showed enrichment in various processes, including plant cell wall modification, rhythmic processes, gibberellin biosynthetic processes, and DNA‐templated transcription (Figure 3D), along with various molecular functions (Figure S4F). Metabolic biosynthesis and related molecular functions were significantly enriched in cluster C4 (Figure S4B,G), while cluster C5 showed significant enrichment for cellular oxidant detoxification‐related processes and enzyme activities (Figure S4C,H). These findings suggest that the DEGs responding to seed vigour are involved in a diverse array of biological pathways.

To further explore the key pathways responding to selfed seed vigour, we performed GO enrichment network analysis on all 1045 overlapping DEGs. This analysis revealed a complex network of interconnected biological processes and molecular functions. Among them, adenine salvage and nucleoside metabolism were central, with connections to numerous other terms including AMP salvage, transcription (DNA‐templated), defence response, carbohydrate metabolic processes, and cell wall‐related processes like xyloglucan metabolism (Figure 3E). To further prioritise critical pathways, we integrated these results with hub gene identification based on regulatory network (*r* > 0.8 and *p* < 0.05) (Figure S5). This identified gene_BSBN01000500.1_000130 (adenine phosphoribosyl transferase 3, *APRT3*), a key enzyme in the adenine salvage pathway (purine metabolism), as the top‐ranked hub gene (Table S11). Therefore, we propose that the adenine salvage pathway may play an important role in the response to the vigour of inbred seeds.

### 3.4 Efficient Purine Utilisation Underlies Enhanced Seed Vigour in Selfed Progeny

**FIGURE 4:**
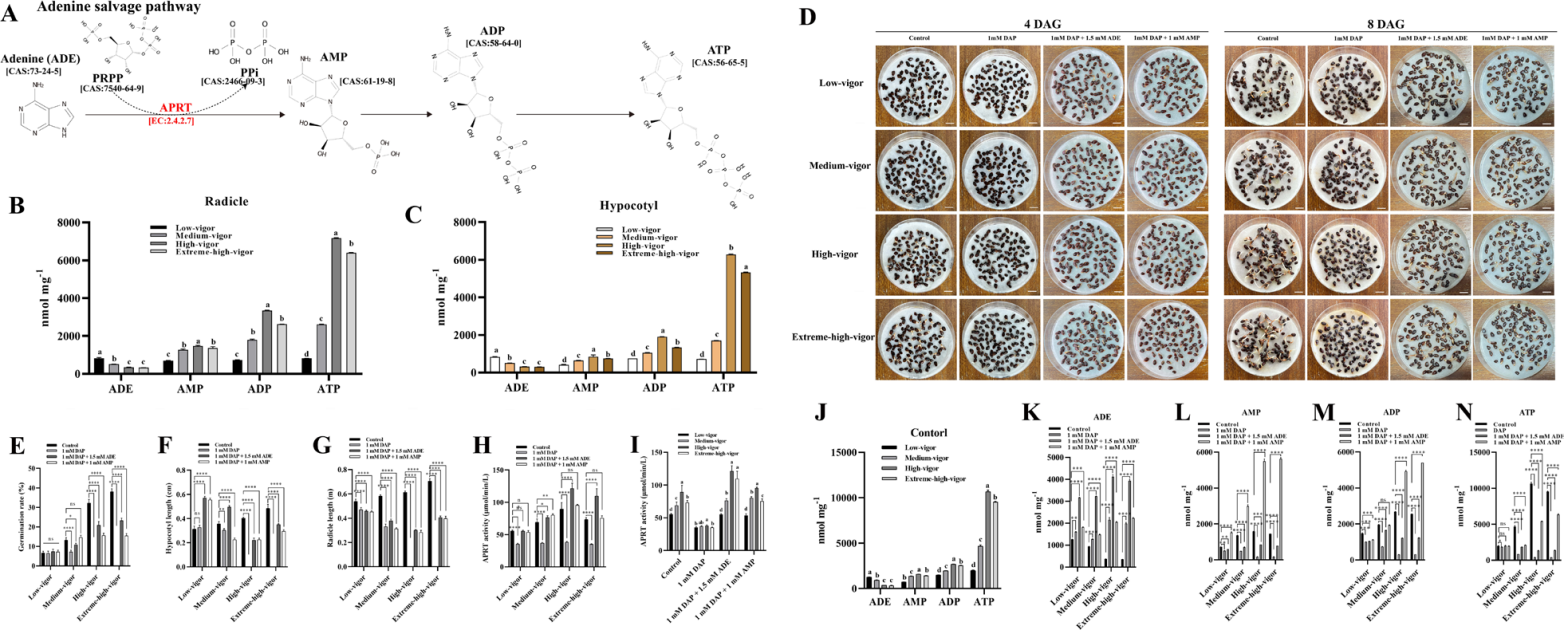
Purine metabolites and adenine phosphoribosyl transferase (APRT) enzyme activity among inbred seeds. (A) Schematic representation of enzymatic reaction catalysed by the APRT protein in adenine salvage pathway. (B) The comparison of ADE, AMP, ADP, and ATP levels in the radicles of inbred and non‐inbred seeds at the 14th DAG. Error bars indicate ± SD (*n* = 3). (C) The comparison of ADE, AMP, ADP, and ATP levels in the hypocotyls of and non‐inbred at the 14th DAG. Error bars indicate mean ± SD (*n* = 3). (D) Dynamic germination of inbred seeds and non‐inbred seeds when treated with water, DAP, DAP+ADE and DAP+AMP. Each experiment was repeated six times with 100 seeds in each replication. The white scale represents 1.0 cm. The germination rate (E), hypocotyl length (F), radicle length (G), APRT enzyme activity (H) of inbred seeds and non‐inbred seeds under treated with water, DAP, DAP+ADE, and DAP+AMP at 8th DAG. Error bars indicate mean ± SD (*n* = 3). (I) The comparison of APRT activity among inbred seeds when treated with water, DAP, DAP+ADE and DAP+AMP at 8th DAG. Error bars indicate mean ± SD (*n* = 3). (J) The comparison of purine metabolites (ADE, AMP, ADP and ATP) among inbred seeds when treated with water, DAP, DAP+ADE and DAP+AMP at 8th DAG. Error bars indicate mean ± SD (*n* = 3). Comparison of ADE (K), AMP (L), ADP (M) and ATP (N) levels in inbred and non‐inbred seeds under different treatments: Water, DAP, DAP+ADE and DAP+AMP at the 8th DAG. Error bars indicate mean ± SD (*n* = 3). Different letters denote statistically significant differences resulting from Duncan's test following one‐way ANOVA. DAG, ADE, ADP, AMP and ATP indicate days after germination, adenine, adenosine diphosphate, adenosine monophosphate, and adenosine triphosphate, respectively. Different letters denote statistically significant differences resulting from Duncan's test following one‐way ANOVA. *p < 0.05, **p < 0.01, ***p < 0.001, ****p < 0.0001 (Student's *t*‐test). PRPP, phosphoribosyl pyrophosphate; AMP, adenosine‐5′‐monophosphate; ADP, adenosine diphosphate; ATP, adenosine triphosphate; PPi, pyrophosphoric acid.

Adenine phosphoribosyl transferase (APRT) is a key enzyme in the adenine salvage pathway, catalysing the conversion of adenine into AMP. This enzyme plays a pivotal role in maintaining the balance of the purine nucleotide pool in plants (Allen et al. 2002) (Figure 4A). To explore whether purine metabolites (AMP, ADP, and ATP) vary among inbred seeds, we performed LC–MS analysis on the radicles and hypocotyls at 14 days after germination. The purine metabolite levels in both radicles (Figure 4B) and hypocotyls (Figure 4C) were highest in high‐vigour seeds, lowest in low‐vigour seeds, and intermediate in medium‐vigour seeds. This trend was consistent with the vigour levels in inbred seeds. However, in extreme‐high‐vigour seeds (non‐inbred seeds), the purine metabolite levels in both the radicles (Figure 4B) and hypocotyls (Figure 4C) were lower than those in high‐vigour seeds, but higher than those in medium‐vigour seeds. However, the levels of adenine metabolites among inbred seeds were highest in low‐vigour seeds, followed by medium‐ and high‐vigour seeds, and lowest in extreme‐high‐vigour seeds, which implies a more efficient utilisation of purine metabolites in seeds with higher vigour. This view was further supported by the ATP/ADP ratio, which was highest in extreme‐high‐vigour seeds, followed by high‐ and medium‐vigour seeds, and lowest in low‐vigour seeds (Figure S6A,B). A similar trend was also observed in the ATP/AMP ratio. Collectively, our results demonstrate that purine metabolism plays a key role in determining seed vigour, with higher‐vigour inbred seeds showing more efficient purine utilisation.

### 3.5 The Adenine Salvage Pathway Is Essential for Seed Germination Under Selfed Conditions

Studies have shown that 6‐diaminopurine (DAP), a purine analogue and APRT inhibitor, effectively suppressed APRT activity, thereby blocking the purine salvage pathway and inhibiting cell growth and proliferation (Victor et al. 1999). To investigate the role of the APRT enzyme in the germination of inbred seeds, we applied the APRT inhibitor DAP and examined its effects on seed germination. Given the potential impact of inhibitor concentration on seed germination rates, we first treated open‐pollinated progeny seeds with a gradient of DAP concentrations, which were characterised as low (10 μmol·L^−1^ and 25 μmol·L^−1^), moderate (50 μmol·L^−1^, and 100 μmol·L^−1^), high (0.5 mmol·L^−1^ and 1 mmol·L^−1^), and extremely high (5 mmol·L^−1^). Germination assays revealed that high concentrations of DAP significantly inhibited seed germination, while extremely high concentrations completely suppressed seed germination (Figure S7A). Specifically, at a concentration of 1 mmol·L^−1^ DAP, seed germination (Figure S7B), radicle length (Figure S7C), and hypocotyl length (Figure S7D) were significantly reduced. Based on these results, 1 mmol/L DAP was identified as an optimal concentration for studying the effects of APRT enzyme inhibition on the germination of inbred seeds.

To investigate the effect of 1 mmol·L^−1^ DAP on seed germination, seeds were treated with this concentration of DAP. On the 4th day after germination (DAG), no seeds from the low‐, medium‐, high‐ and extreme‐high‐vigour groups treated with 1 mmol/L DAP had successfully germinated. In contrast, varying numbers of germinated seeds were observed in all control groups (Figure 4D). By the 8th DAG, the addition of DAP had no significant effect on the germination rate (Figure 4E) or on hypocotyl length (Figure 4F) in low‐vigour seeds, but it significantly reduced the radicle length (Figure 4G). In medium‐vigour seeds, the DAP treatment resulted in a germination rate about half that of the control group (Figure 4E), and both radicle and hypocotyl growth were significantly inhibited (Figure 4F,G). In contrast, high‐ and extreme‐high‐vigour seeds did not germinate at all, suggesting that DAP induced embryo lethality in these seeds (the endosperm and embryo of these high‐ and extreme‐high‐vigour seeds had softened and disintegrated at 10th DAG). Surprisingly, the addition of adenine (ADE) at 1.5 mmol·L^−1^ to the sterile water containing DAP (1 mmol·L^−1^) alleviated the inhibitory effects of DAP on medium‐, high‐ and extreme‐high‐vigour seeds (Figure 4D). This effect was noticeable on the 4th day of initial germination. By the 8th DAG, the germination rate of high‐ and extreme‐high‐vigour seeds partially recovered to about half of the control group (Figure 4E), and medium‐vigour seeds also showed some recovery. Additionally, the simultaneous presence of DAP and ADE significantly increased the hypocotyl length of low‐ and medium‐vigour seeds compared to the control group (Figure 4F). However, despite the partial recovery in germination rates in high‐ and extreme‐high‐vigour seeds, both their hypocotyl (Figure 4F) and radicle (Figure 4G) lengths remained significantly reduced. To further explore these findings, AMP (1 mmol·L^−1^), a direct product of APRT enzyme‐catalysed substrate synthesis, was included in the DAP solution. The results were similar to those observed with ADE and DAP; the addition of AMP partially recovered the germination and growth of medium‐ and high‐vigour seeds (Figure 4D–G). This suggests that both ADE and AMP might positively influence seed germination. To confirm this hypothesis, inbred seeds were treated with a suitable concentration of ADE (100 μmol·L^−1^), based on a gradient concentration experiment conducted on open‐pollinated seeds (Figure S8). This concentration of ADE did not affect germination but promoted subsequent radicle and hypocotyl growth (Figure S9). This promotive effect was most pronounced in high‐vigour seeds, followed by extreme‐high‐vigour seeds, and was least notable in low‐vigour seeds. Collectively, these results suggest that the APRT inhibitor DAP significantly suppressed germination in high‐ and extreme‐high‐vigour seeds, but permits the germination of low‐vigour seeds. The response of medium‐vigour seeds lies between that of high‐ and low‐vigour seeds. Additionally, the inclusion of ADE or AMP positively affects the germination and growth of seeds, particularly the seeds with high‐ and extreme‐high‐vigour.

DAP is known to effectively inhibit APRT activity. Thus, we examined the APRT activity in both inbred and non‐inbred seeds treated with DAP, ADE and AMP. Our results showed that treatment with DAP significantly inhibited APRT activity in low‐, medium‐, high‐, and extreme‐high‐vigour seeds (Figure 4H). However, in the groups treated with both ADE and DAP, APRT activity was restored in all vigour levels of seeds, even exceeding the activity levels observed in the control group. A similar reversal of APRT inhibition was observed in seeds treated with both AMP and DAP. When comparing inbred seeds of various vigour levels, we found that in the water treatment, higher vigour seeds exhibited higher APRT activity (Figure 4I). Non‐inbred seeds, however, followed a different pattern, with their APRT activity being second only to that of high‐vigour inbred seeds. After DAP application, APRT activity was significantly reduced in both inbred and non‐inbred seeds. The addition of either ADE or AMP activated APRT activity in inbred and non‐inbred seeds. This activation effect was more pronounced in seeds with higher vigour. To investigate whether these changes in APRT activity were linked to differences in purine metabolites, we conducted an analysis of purine metabolites. In the control (8th DAG), concentrations of AMP, ADP and ATP were highest in high‐vigour seeds (Figure 4J), followed by medium‐vigour and extreme‐high‐vigour seeds, with the lowest concentrations observed in low‐vigour seeds. This trend mirrored seed vigour levels in inbred seeds, but was different in non‐inbred (extreme‐high‐vigour) seeds, which showed second‐highest levels of these purine metabolites. However, the lowest concentration of ADE was observed in high‐ and extreme‐high‐vigour seeds, while the highest concentration was found in low‐vigour seeds, with medium‐vigour seeds showing intermediate levels. When treated with DAP, concentrations of AMP, ADP, and ATP were significantly decreased in all seed types compared to the control (Figure 4L–N). The addition of ADE or AMP partially recovered these purine metabolite levels. However, ATP levels recovered to a lesser extent than ADP levels (Figure 4M,N). These results suggest that there are differences in the sensitivity of APRT enzyme activity among inbred seeds, as well as non‐inbred seeds. Furthermore, elevated levels of purine metabolites promote seed germination vigour.

### 3.6 *ClAPRT3* Promotes Seed Germination by Enhancing Purine Salvage and Activating Cell Wall–Loosening Genes

**FIGURE 5:**
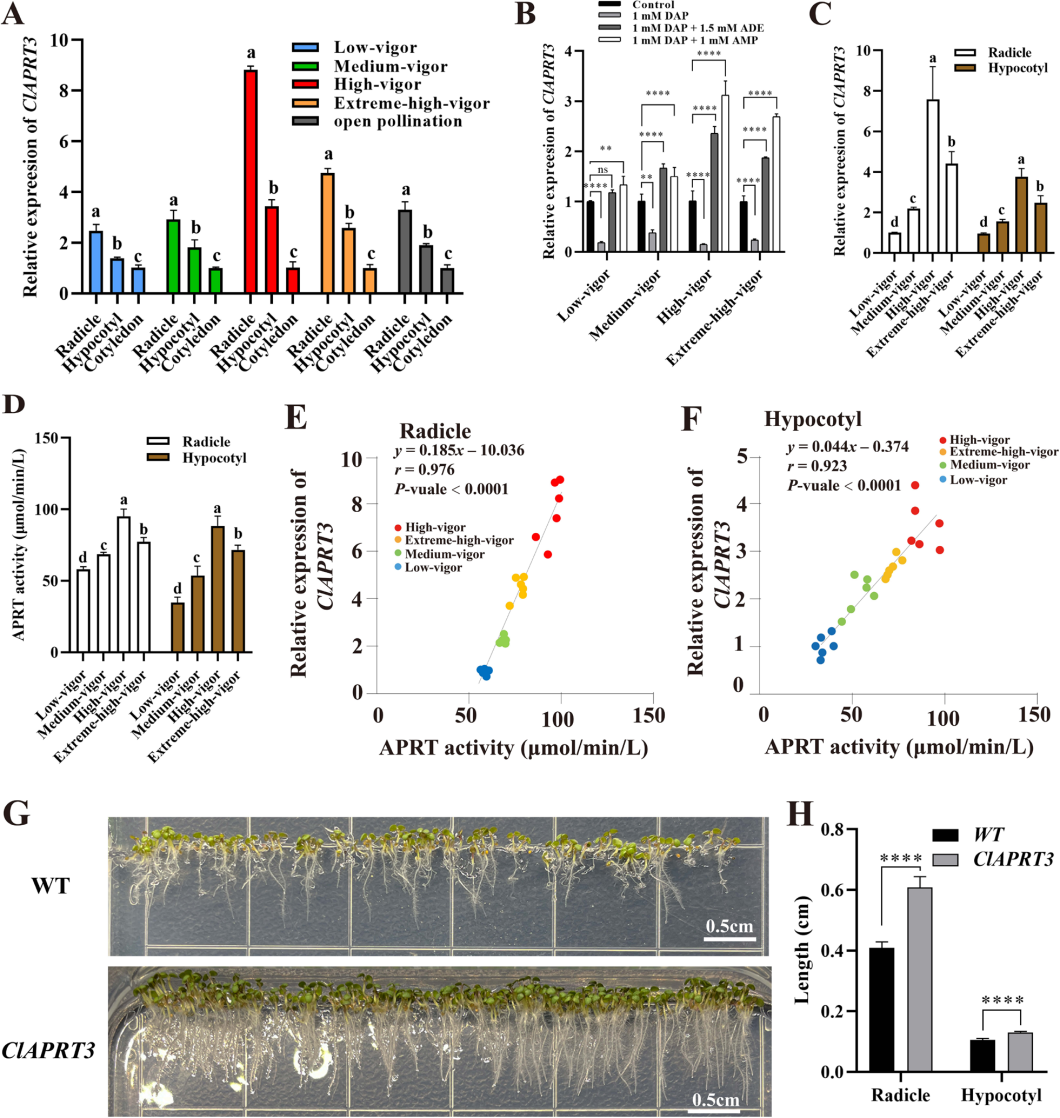
Analyses of *ClAPRT3* expression and adenine phosphoribosyl transferase (APRT) enzyme activity. (A) Tissue‐specific expression analyses of *ClAPRT3* in radicle, hypocotyl, and cotyledon. Error bars indicate ± SD (*n* = 3). (B) Relative expression of *CLAPRT3* of inbred and non‐inbred seeds when treated with water, DAP, DAP+ADE, and DAP+AMP at 8th DAG. Error bars indicate mean ± SD (*n* = 3). (C) Comparison of relative expression of *CLAPRT3* in inbred and non‐inbred seeds. Error bars indicate mean ± SD (*n* = 3). (D) Comparison of APRT enzyme activity in inbred and non‐inbred seeds. Error bars indicate ± SD (*n* = 3). Linear regression analysis was performed between enzymatic activities of APRT and relative expression of *ClAPRT3* in radicles (E) and hypocotyls (F). Error bars indicate mean ± SD (*n* = 6). (G) Phenotypic characteristic between wild‐type (WT) and 35S::*ClAPRT3 Arabidopsis* seedlings on the 5th day after germination. (H) Comparison of radicle and hypocotyl length between WT and 35S::*ClAPRT3 Arabidopsis* seedlings on the 5th day after germination. Different letters denote statistically significant differences resulting from Duncan's test following one‐way ANOVA. DAG, ADE, ADP, AMP and ATP indicate days after germination, adenine, adenosine diphosphate, adenosine monophosphate and adenosine triphosphate, respectively. Different letters denote statistically significant differences resulting from Duncan's test following one‐way ANOVA. **p < 0.01, ****p < 0.0001 (Student's *t*‐test).

The differential levels of APRT enzyme activity and purine metabolites observed among inbred seeds suggest that they may be regulated by the *APRT* gene. Here, we identified a hub gene, *APRT3*, from the regulatory network. *APRT3* is a member of the APRT family that shows high homology to *AtAPT3* (AT4G22570.1) and *AtAPT4* (AT4G12440.2) in *Arabidopsis thaliana* (Figure S10). We hypothesized that *ClAPRT3* (referred as Chinese fir *APRT3*) might play an important role in regulating seed vigour through purine metabolism. To explore this, we first examined the expression patterns of the *ClAPRT3* gene in different tissues of inbred progenies. Interestingly, we found that the expression level of *ClAPRT3* in the radicle and hypocotyl was consistently higher than in the cotyledon across all inbred progenies (Figure 5A). Moreover, the transcript level of *ClAPRT3* was significantly inhibited in both inbred and non‐inbred seeds treated with DAP (Figure 5B). However, the addition of either ADE or AMP restored the expression level of *ClAPRT3* in both inbred and non‐inbred seeds. In inbred seeds, the expression level of *ClAPRT3* in the radicles and hypocotyls increased with higher seed vigour (Figure 5C). In contrast, non‐inbred seeds exhibited a different pattern, with *ClAPRT3* expression being second‐highest. The same trend was observed in the APRT enzyme activity of both the radicles and hypocotyls (Figure 5D). To further investigate the relationship between *ClAPRT3* expression and APRT enzyme activity, we performed a linear regression analysis. Interestingly, a strong positive correlation was observed between *ClAPRT3* expression and APRT enzyme activity in both radicle (*r* = 0.976, *p* < 0.0001) (Figure 5E) and hypocotyl (*r* = 0.923, *p* < 0.0001) (Figure 5F). To further explore the roles of *ClAPRT3* in seed germination, we generated a transgenic *A. thaliana* line expressing *35S::ClAPRT3* (Figure 5G). Overexpression of *ClAPRT3* significantly promoted radicle and hypocotyl length (Figure 5H). Furthermore, overexpression of *ClAPRT3* led to a significant increase in the levels of purine metabolites, including ADE, AMP, ADP and ATP, compared to the wild‐type (WT) (Figure S11A). The ATP/ADP ratio was also markedly elevated in the transgenic seedlings (Figure S11B), indicating a more favourable cellular energy status. These findings suggest that *ClAPRT3* overexpression promotes purine salvage and energy homeostasis, thereby enhancing seed vigour and supporting early seedling establishment.

Cell‐wall‐loosening related *EXP* genes have been identified as promoters of seed germination by facilitating cell elongations (Huang et al. 2024). In our study, we identified a similar gene, gene_BSBN01001351.1_000005 (designated as *CLEXPL1* in *C. lanceolata* ), which encodes an expansion‐like a1‐related protein and ranks among the top 50 genes in the regulatory network (Table S11). Transcript analysis revealed that *CLEXPL1* expression levels were highest in high‐vigour seeds, followed by extreme‐high and high‐vigour seeds, with the lowest expression observed in low‐vigour seeds (Figure S12A). However, *CLEXPL1* expression was significantly suppressed in both inbred and non‐inbred seeds treated with DAP (Figure S12B). Partially recovery of *CLEXPL1* expression was observed in seeds treated with DAP and ADE. These effects were consistent across both inbred and non‐inbred seeds. The expression profiles of *ClAPRT3* and *ClEXPL1* under different treatments showed a similar trend across inbred and non‐inbred seeds of varying vigour levels (Figure S13), suggesting a potential regulatory association. Furthermore, this similar trend was also observed between the APRT activity and the expression profiles of *ClEXPL1* (Figure S14), indicating that purine metabolism—particularly the adenine salvage pathway—may influence the expression of genes involved in cell wall loosening, which are crucial for seed germination and early seedling growth. These findings suggest that the *ClAPRT3*‐mediated adenine salvage pathway enhances purine metabolite levels, which in turn upregulates the expression of cell wall loosening‐related genes, thereby improving seed germination vigour.

### 3.7 *ClMYB23* Negatively Regulates the Expression of *ClAPRT3*

**FIGURE 6:**
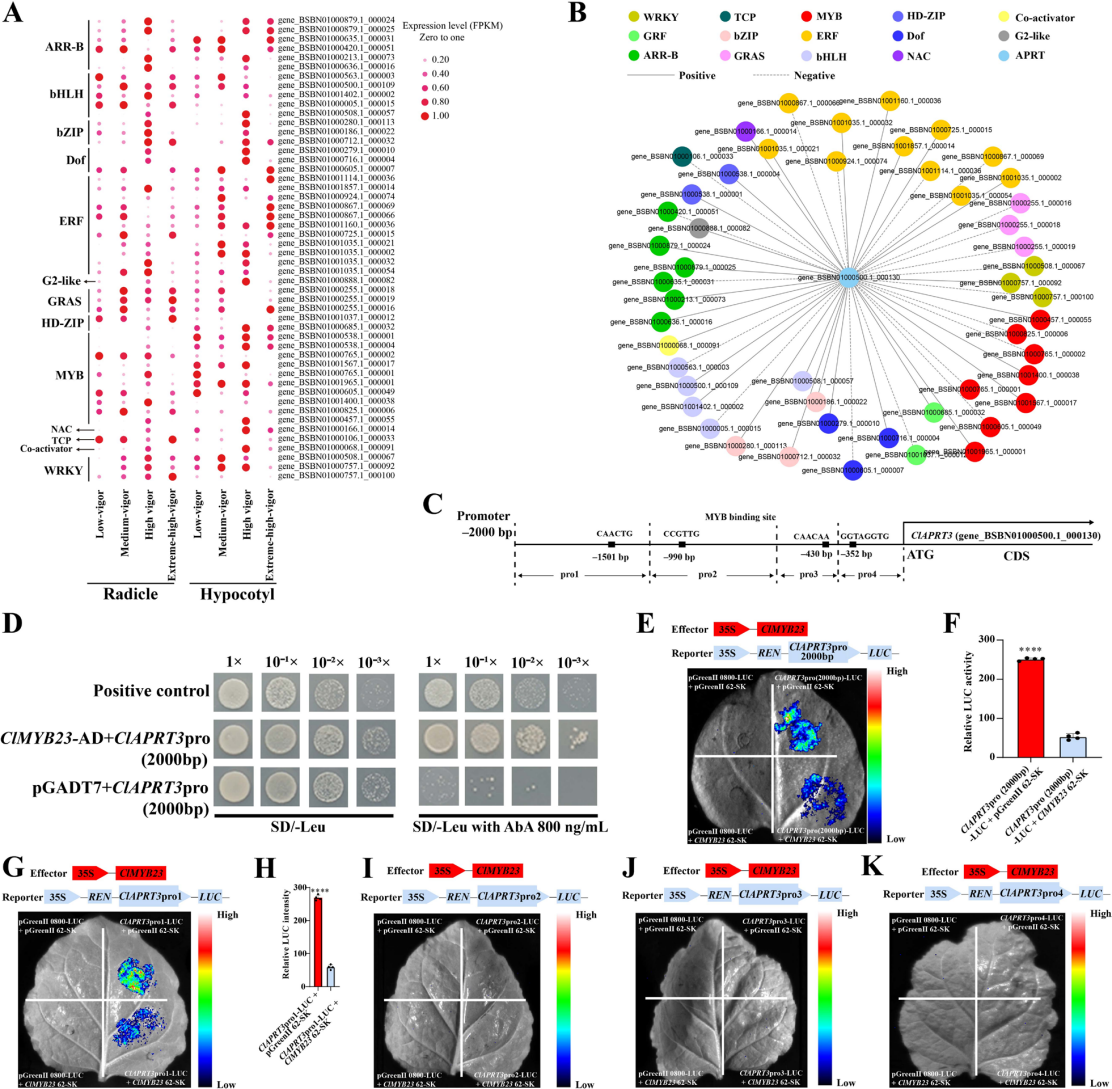
Expression profiles of candidate transcription factor (TFs) and ClMYB23 (gene_BSBN01000765.1_000002) interacts with *ClAPRT3* (gene_BSBN01000500.1_000130). (A) The heatmap shows the expression patterns of TFs. (B) TFs linked to *ClAPRT3*. (C) Potential MYB binding sites in the *ClAPRT3* gene promoter (2000 bp), including pro1, pro2, pro3, and pro4. (D) Yeast one‐hybrid assays between *ClMYB23* and the 2000 bp promoter of *ClAPRT3*. (E) Dual‐luciferase assays of *ClMYB23* with *ClAPRT3* promoter (*ClAPRT3*pro 2000 bp). (F) Relative luciferase activities for co‐expressed *ClAPRT3*pro‐LUC + empty vector (pGreenII 62‐SK) and *ClAPRT3*pro‐LUC + *ClMYB23*‐SK. (G) Dual‐luciferase assays of *ClMYB23* with *ClAPRT3* promoter (pro1). (H) Relative luciferase activities for co‐expressed *ClAPRT3*pro1‐LUC + empty vector (pGreenII 62‐SK) and *ClAPRT3*pro1‐LUC + *ClMYB23*‐SK. (I) Dual‐luciferase assays of *ClMYB23* with *ClAPRT3* promoter (pro2). (J) Dual‐luciferase assays of *ClMYB23* with *ClAPRT3* promoter (pro3). (K) Dual‐luciferase assays of *ClMYB23* with *ClAPRT3* promoter (pro4). Combinations containing empty vector were used as negative controls. Asterisks indicate statistically significant differences (Student's *t*‐test; **p < 0.01, ****p < 0.0001, mean ± SD). Error bars indicate a standard deviation of three biological replicates. (G) On MS medium, *A. thaliana* lines expressing *35S::ClAPRT3* exhibited longer radicle and hypocotyl lengths compared to the WT (Five‐day‐old seedlings). Values represent the mean ± SD. Error bars indicate a standard deviation of three biological replicates with 100 seeds in each replication. ****p < 0.0001 (Student's *t*‐test).

Transcription factors (TFs) serve as master regulators of gene expression (Omidbakhshfard et al. 2015). In our study, we observed distinct expression patterns of *ClAPRT3* in both radicles and hypocotyls (Figure 5A–C) of inbred seeds, suggesting potential regulation by TFs. Among the overlapping DEGs (1045) identified (Figure 3A), we found 50 TFs spanning 13 families, including ARR‐B, bHLH, bZIP, Dof, ERF, G2‐like, GRAS, HD‐ZIP, MYB, NAC, TCP, WRKY and one transcription co‐activator (MBF1). Heatmap analysis showed significant variability in the expression levels of these TFs among inbred seeds (Figure 6A). Correlation analysis of gene expression trends identified gene_BSBN01000765.1_000002 as a potential key upstream regulator of *ClAPRT3*. The transcriptional regulatory subnetwork for *ClAPRT3* further indicated a negative correlation between gene_BSBN01000765.1_000002 and *ClAPRT3* expression (Figure 6B). Across both radicle and hypocotyl tissue, gene_BSBN01000765.1_000002 exhibited the highest expression in low‐vigour seeds, followed by medium and high‐vigour seeds, with the lowest expression in extreme‐high‐vigour seeds (Figure S15). To classify gene_BSBN01000765.1_000002, we cloned the gene and performed protein alignment using the NCBI database. The analysis revealed that gene_BSBN01000765.1_000002 shares 62.65% similarity with *MYB23* (XP_057854396.1) from *Cryptomeria japonica* , a member of the Cupressaceae family (Figure S16). Consequently, we named this gene *ClMYB23* in Chinese fir. Furthermore, analysis of the *ClAPRT3* promoter using the PlantCARE online platform identified multiple MYB binding sites (pro1, pro2, pro3 and pro4) (Figure 6C).

Based on these above findings, we hypothesized that *ClMYB23* negatively regulates the expression of *ClAPRT3*. To test this hypothesis, we performed a yeast one‐hybrid assay and found that *ClMYB23* interacts with the promoter of *ClAPRT3* (Figure 6D). To further examine whether *ClMYB23* directly represses the transcription of *ClAPRT3*, we conducted a dual‐luciferase reporter assay. For this, we constructed the *35S*::*ClMYB23* effector and *ClAPRT3*::LUC reporter plasmids, and the plasmid‐containing *Agrobacterium* was infiltrated into the epidermal cells of *Nicotiana benthamiana* leaves. The results showed that the activity of the *ClAPRT3*::LUC reporter gene was suppressed in the presence of the *35S*::*ClMYB23* effector (Figure 6E). Relative LUC activity was significantly lower when the *35S*::*ClMYB23* effector was present compared to its absence, providing strong evidence that *ClMYB23* directly inhibits *ClAPRT3* expression (Figure 6F). These results confirmed that *ClMYB23* negatively regulates the transcription of *ClAPRT3*. To determine which region of the *ClAPRT3* gene promoter mediates this regulatory relationship, we performed segmental dual‐luciferase assays on individual regions of the *ClAPRT3* promoter (pro1, pro2, pro3 and pro4). Our experiments revealed that pro1 was the only region capable of inducing the interaction between ClMYB23 and *ClAPRT3* (Figure 6G). Relative LUC activity further confirmed that ClMYB23 specifically inhibits *ClAPRT3* through the pro1 region (Figure 6H). No interactions were detected between ClMYB23 and *ClAPRT3* promoter regions pro2 (Figure 6I–K), pro3 or pro4. To further determine whether the transcriptional expression differences of *ClAPRT3* in inbred progenies are caused by variations in the promoter sequence, we cloned the *ClAPRT3* promoter sequences from different progenies and performed sequence alignments. The results showed no mutations in the MYB binding motifs (Figure S17). This indicates that the differential expression of *ClAPRT3* is not caused by deletion or mutation of the MYB binding site, but rather results from complex transcriptional regulatory mechanisms, including the differential expression of ClMYB23 (Figure S15).

### 3.8 Characterisation of *ClAPRT3* Coding Sequence From Different Inbred Progeny

**FIGURE 7:**
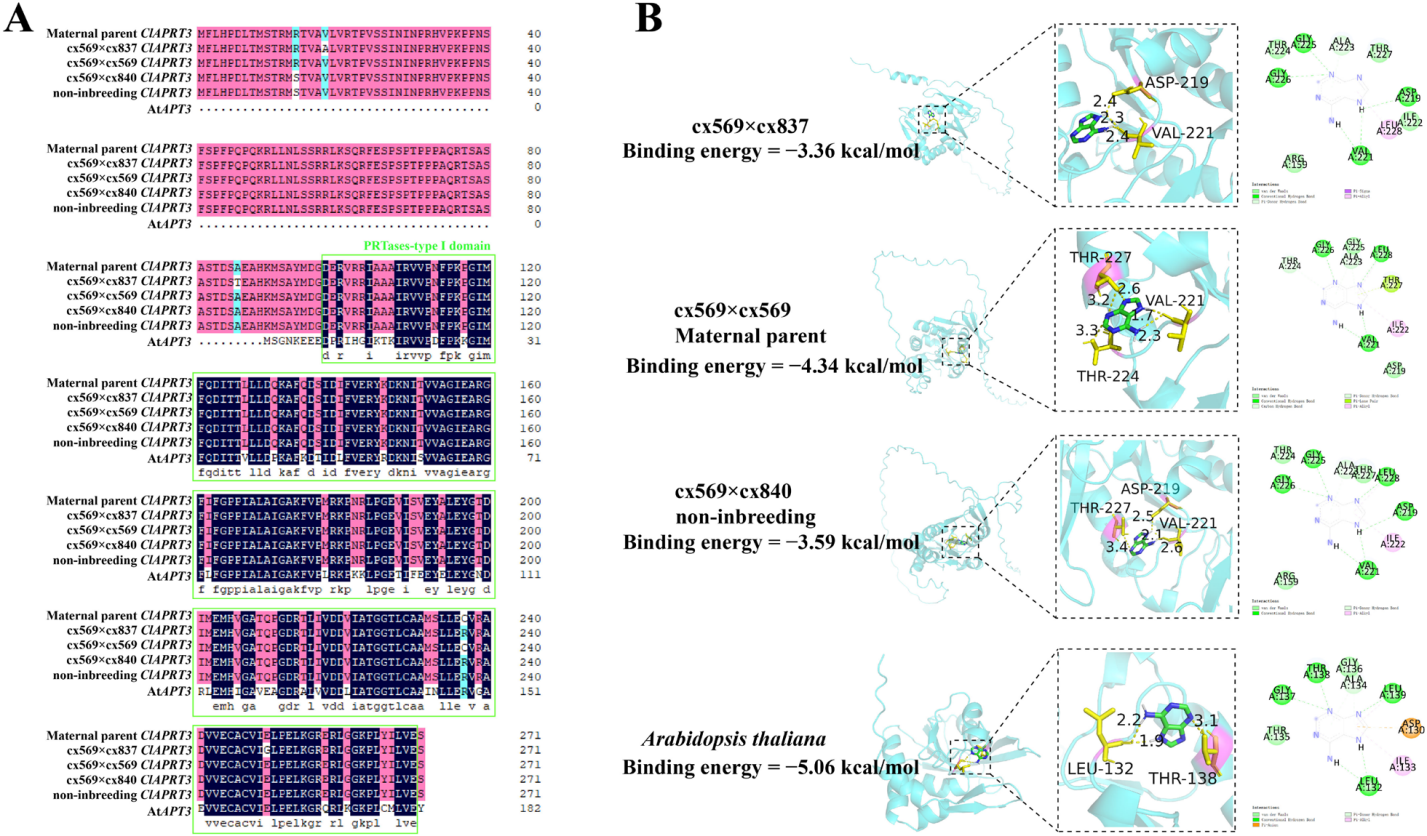
Coding sequence characterisation of *ClAPRT3* from different inbred progeny in *Cunninghamia lanceolata* . (A) Sequence alignment of *ClAPRT3* and their ortholog in *Arabidopsis Thaliana* . (B) Molecular docking between receptor ClAPRT3 and its ligand (Adenine).

The coding sequence (CDS) represents the protein‐coding region, where sequence variations can lead to changes in protein structure and subsequently impact protein activity. To investigate this, we cloned the *ClAPRT3* CDS from different inbred progeny. Sequence alignment showed a nonsynonymous mutation in the *ClAPRT3* CDS among the inbred progeny (Figure 7A). We hypothesised that these CDS sequence variations might alter the protein structure and active sites. To test this hypothesis, we conducted molecular docking simulations between the receptor protein (*ClAPRT3*) and the ligand (adenine) to evaluate binding and identify key active sites. In the low‐vigour progeny (cx569 × cx837), substitutions of ASP at position 219 and VAL at position 221 in the receptor site resulted in a higher binding energy (−3.36 kcal/mol) compared to medium‐vigour (cx569 × cx569) and maternal parent, which had VAL at position 221, THR at position 224, and THR at position 227 (−4.34 kcal/mol) (Figure 7B). However, for high‐vigour progeny (cx569 × cx840) and non‐inbred progeny showed binding sites that included VAL at position 221, ASP at position 219, and THR at position 227, with a binding energy of −3.59 kcal/mol. Comparative analysis of ClAPRT3‐ligand sites across inbred progeny revealed that the ClAPRT3 protein in selfed progeny and the maternal retained an active site at position 227 (THR), whereas low‐, high‐, and extreme‐high‐vigour progeny lacked an active position 224 (THR). Notably, the lowest binding energy (−5.06 kcal/mol) was observed for the homologous APT3 protein in *Arabidopsis*, with a key binding site at position 138 (THR), which corresponds to position 227 of *C. lanceolata* CIAPRT3 (see Figure 7B). These findings suggest that position 227 may act as a crucial binding site, contributing to the structural stability of the complex.

## 4 Discussion and Conclusion

Selfing often causes severe inbreeding depression in the plant life cycle, especially at early stages (Collin et al. 2009). In this study, we observed that selfed seed quality, including parameters such as the cone‐seed rate, seed soundness, thousand kernel weight, astringent seed rate, empty seed rate, and embryo abortion rate as well as germination vigour (germination potential, germination rate), was significantly lower than those of non‐inbred progeny. These findings align with the general consensus that selfing often leads to inbreeding depression, reducing offspring vigour (Kephart et al. 1999; Shibata et al. 2018; Rymer et al. 2015; Dieterich Mabin et al. 2021). Interestingly, despite these expected effects, selfed progeny did not always exhibit the lowest vigour when compared to certain inbred progenies (e.g., cx569 × cx837 and cx569 × cx865) (Figure 1). A comprehensive evaluation further revealed that selfed seeds exhibited moderate vigour (Table S7), with radicle and hypocotyl lengths even exceeding those of some backcrossed seeds (e.g., cx569 × cx837) (Figure 2). This contrasts with the perspective of Charlesworth and Willis (2009), who argued that selfed progeny generally exhibit lower vigour than other inbred progeny due to greater parental relatedness. However, other studies have shown that certain tree species or genotypes exhibit low levels of inbreeding depression in particular traits (El‐Kassaby et al. 1994; Wu et al. 2016; Murren and Dudash 2012; Cuénin et al. 2019). Recently, Shojaiefar et al. (2022) demonstrated that selfing produced high‐quality seeds with higher oil content than open‐pollinated seeds in *Foeniculum vulgare* . These results indicate that selfing does not necessarily produce the least vigorous progeny. In other words, while selfing generally results in reduced vigour, our findings show that selfed progeny can outperform certain inbred backcrosses, suggesting that inbreeding depression is not uniformly more severe in selfed progeny.

Inbreeding depression has long been attributed to classical genetic mechanisms such as the expression of deleterious recessive alleles and loss of heterozygote advantage (Zhang et al. 2019), or reduced recombination frequency in certain genomic regions (McMullen et al. 2009). However, emerging evidence highlights epigenetic regulation as a key contributor to inbreeding effects (Han et al. 2021), which may in turn disrupt gene regulatory networks and disturb metabolic balance (Achrem et al. 2023). In this study, we identified *ClAPRT3*, a key enzyme in the adenine salvage pathway of purine metabolism, as a hub gene associated with seed vigour maintenance during selfing in *Cunninghamia lanceolata* . GO enrichment and network analyses revealed that adenine salvage (purine metabolism) connects to multiple biological processes, including stress response, transcriptional regulation and cell wall modification (Figure 3E). This aligns with previous reports showing that inbreeding primarily impacts stress‐related and metabolic genes across species (Kristensen et al. 2010; Menzel et al. 2015). Species‐specific responses have also been observed; for example, Liu et al. (2018) found that auxin‐response and synthesis pathways were differentially expressed in inbreeding depression lines of *Brassica rapa* . Together, these findings point out that selfing induces both conserved and context‐specific molecular responses, underscoring the complex regulation of inbreeding depression.

Adenine salvage, a critical component of purine metabolism, contributes significantly to seed germination and plant growth (Price and Murray 1969; Anderson 1977; Lee and Moffatt 1994; Sukrong et al. 2012; Bray 2017). Consistent with this, the purine metabolism pathway was activated during germination (Wang et al. 2022), whereas reduced salvage activity has been shown to impair normal growth (Ashihara et al. 2018). In this study, purine metabolites (AMP, ADP, ATP) levels were significantly lower in low‐ to medium‐vigour selfed seeds compared to extremely high‐vigour non‐inbred controls (Figure 4B,C,J). Interestingly, high‐vigour inbred seeds showed elevated levels of purine metabolites (Figure 5D), yet the ATP/ADP ratio remained lower than that of non‐inbred seeds (Figure S6), indicating lower energy utilisation efficiency. These results support a view that inbred individuals generally exhibit lower metabolic efficiency (Kristensen et al. 2006; Ayroles et al. 2009). However, this is not completely consistent with the opinion that inbreeding generally upregulates genes involved in metabolic processes (Kristensen et al. 2006; Menzel et al. 2015). For example, the expression level of *ClAPRT3* in selfed seeds was consistently lower than that in non‐inbred seeds (Figure 5C). Another interesting observation was that APRT, the key enzyme in the adenine salvage pathway, appeared to influence germination responses to its specific inhibitor, 6‐diaminopurine (DAP). Germination was markedly inhibited in high‐ and extreme‐high‐vigour seeds under DAP treatment, while low‐vigour seeds were less affected (Figure 4D). Medium‐vigour seeds (i.e., selfed progeny) exhibited intermediate germination performance. DAP, an adenine analogue, is converted into toxic nucleotides in APRT+ cells, leading to cell death, whereas APRT‐ cells are less affected because they rely on the de novo pathway for AMP synthesis and do not take up the toxic metabolites (Schaff 1994). These results suggest that high‐vigour seeds likely retain functional APRT activity (APRT+), while low‐vigour seeds tend to be APRT‐deficient (APRT‐), and the intermediate sensitivity of selfed seeds reflects a partial retention of APRT function. APRT may serve as a potential selectable marker for identifying inbreeding depression in plants, as it has been successfully applied in animal (Schaff et al. 1990) and bacterial systems (Taylor et al. 1985), and its functionality has also been demonstrated in plants (Moffatt and Somerville 1988). Collectively, these results suggest that reduced metabolic efficiency, downregulation of related genes, and a compromised energy state may contribute to vigour loss. In other words, the maintenance of seed vigour in selfed progeny likely depends on maintaining metabolic efficiency and ensuring adequate energy supply, in part through sustained adenine salvage.

Another key adenine metabolite, adenine (the substrate of APRT) shows an inverse correlation with seed vigour, with higher levels detected in low‐vigour seeds (Figure 4B,C,J). There are two possible explanations for this pattern. First, reduced APRT activity in low‐vigour seeds may lead to limited substrate utilisation. Second, adenine may act as a stress‐responsive molecule. Sukrong et al. (2012) reported that decreased *APRT1* expression in *Arabidopsis thaliana* led to reduced APRT enzymatic activity, elevated adenine levels, enhanced growth and activation of the antioxidant system. Studies have shown that adenine nucleotides also affect ROS production by directly regulating mitochondrial components (Jardim‐Messeder et al. 2022). However, in this study, we found that ROS levels in selfed seeds were significantly lower than those in low‐vigour seeds but higher than those in high‐ and extreme‐high‐vigour seeds (data not shown). A similar trend was observed for peroxidase (POD) activity, a key ROS‐scavenging enzyme, which was intermediate in selfed seeds, high in low‐vigour seeds, and low in high‐vigour seeds (data not shown). A plausible explanation for this phenomenon is that gene and protein regulation may be more complex under inbreeding conditions, and the observed changes likely reflect a physiological adjustment to inbreeding‐induced stress. Notably, adenine can also lead to deleterious, growth‐inhibitory effects if elevated beyond a threshold level (Sukrong et al. 2012). Based on these results, we hypothesise that reduced APRT activity and increased endogenous adenine levels in selfed seeds may represent an adaptive response to inbreeding‐induced stress.

Linear regression analysis revealed a strong correlation between *ClAPRT3* expression and APRT enzyme activity in both radicle (*r* = 0.976, *p* < 0.0001; Figure 5E) and hypocotyl (*r* = 0.923, *p* < 0.0001; Figure 5F), indicating that *ClAPRT3* expression is a reliable indicator of APRT activity. This tight correlation supports its role in adenine salvage, consistent with its similarity to *Arabidopsis AtAPT3* (Allen et al. 2002; Figure S10). *ClAPRT3* overexpression enhances purine salvage and energy efficiency (Figure S11), thereby improving seed vigour and promoting early seedling establishment, which supports the view that energy metabolism plays a crucial role in seed germination. Moreover, radicle elongation was significantly enhanced in *Arabidopsis* overexpression lines, supporting the use of radicle length as a reliable indicator of seed vigour. Studies have shown that EXPANSINs play an important role in seed germination by promoting cell elongation (Marowa et al. 2016; Cosgrove 2024; Huang et al. 2024). In the downstream pathway of purine metabolism, both *ClAPRT3* expression and APRT enzyme activity were positively correlated with *ClEXPL1*, a homologue of the cell wall‐loosening EXPANSIN gene EXP4 (Huang et al. 2024). These results support the hypothesis that *ClAPRT3* contributes to seed vigour. Additionally, overexpression of EXPANSIN genes has been reported to influence antioxidant enzyme activity, particularly that of cell wall‐bound peroxidases (Han et al. 2015). Thus, we speculate that *ClEXPL1* not only directly regulates cell elongation, but also affects the antioxidant system. Collectively, the maintenance of seed vigour under selfing is likely mediated by enhanced purine metabolite levels via the adenine salvage pathway, which may subsequently upregulate cell elongation‐related genes and promote seed germination. How the adenine salvage pathway is functionally linked to EXPANSINs, and how both contribute to ROS modulation and seed vigour maintenance under selfing, remains unclear and may provide valuable insights for future investigations into the mechanisms underlying inbred seed performance.

Interestingly, our findings demonstrate that ClMYB23 acts as a negative regulator of *ClAPRT3* expression, although no mutations were detected in the MYB‐binding sites of its promoter in either inbred or non‐inbred progeny. Notably, to the best of our knowledge, MYB‐mediated regulation of the adenine salvage pathway has not been previously reported (Liu et al. 2023). This suggests that *ClAPRT3* expression differences likely result from upstream transcriptional regulation or sequence variation in other regions of the *ClAPRT3* promoter rather than MYB‐binding motifs variation. In addition, nonsynonymous mutations in the *ClAPRT3* coding sequence were identified among inbred progeny. These mutations altered protein‐ligand binding energy, with low‐vigour progeny (e.g., cx569 × cx837) showing higher binding energy than high‐vigour ones (e.g., cx569 × cx840). Selfed progeny displayed lower binding energy, similar to the maternal parent. Notably, a specific amino acid substitution (THR227) found in selfed and high‐vigour progeny, as well as in *Arabidopsis* with low binding energy, may contribute to more stable enzyme‐substrate interaction and enhanced catalytic efficiency. Thus, allelic variation in *ClAPRT3* could partly explain the variation in APRT activity and purine metabolism in inbred seeds, and may underlie the individual differences observed in inbreeding depression (Schultz and Willis 1995).

This study has several limitations that warrant further investigation. While amino acid variations may explain differences in catalytic efficiency, APRT activity could also be influenced by physiological factors such as substrate availability and interactions with other metabolic enzymes. In addition, the absence of replicated experiments using seeds with comparable vigour and reciprocal crosses limits the generalizability of our findings. The role of APRT‐mediated purine homeostasis in other developmental stages and species remains unclear. Future research should explore whether similar purine salvage mechanisms are conserved across self‐ and cross‐pollinated species.

**FIGURE 8:**
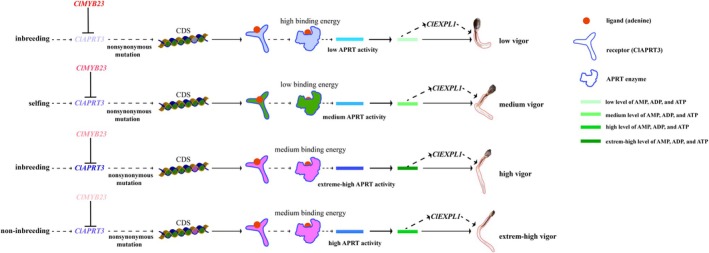
A proposed model illustrating the maintenance of vigour in selfed seeds. Inbreeding led to variation in the expression level of *ClAPRT3*, which was negatively regulated by ClMYB23. The darker the red colour of the *ClAPRT3* font, the higher its expression level; similarly, the darker the blue colour of the *ClMYB23* font, the higher its expression level. Inbreeding also caused variation in the coding sequence of *ClAPRT3*, resulting in altered binding energy between the ligand (adenine) and the receptor (*ClAPRT3*). Overall, a lower binding energy between the ligand and the receptor corresponds to higher APRT enzyme activity, leading to increased levels of purine metabolites (AMP, ADP and ATP), which in turn upregulate the expression of the cell‐wall‐loosening related gene *EXPL1*. This ultimately promotes radicle and hypocotyl elongation during seed germination.

In conclusion, our study demonstrates that *ClAPRT3* plays a critical role in maintaining seed vigour during selfing. We propose a model in which *ClAPRT3* modulates purine metabolite levels (AMP, ADP and ATP) to support seed vigour under inbreeding conditions (Figure 8). The expression of *ClAPRT3* is negatively regulated by ClMYB23, with stronger repression observed in seeds with higher vigour. Inbreeding led to nonsynonymous mutations in the coding sequence (CDS) of *ClAPRT3*, altering the binding energy between the protein and its substrate, adenine, potentially affecting protein stability and enzymatic efficiency. Notably, higher seed vigour was associated with increased purine metabolite accumulation, which may enhance the expression of cell wall‐loosening genes, thereby promoting radicle and hypocotyl elongation.

## Author Contributions

Yun Li, Huiquan Zheng, and Yousry A. El‐Kassaby conceived and designed the experiments. Ye Zhao provided critical guidance for experiment implementation during the major revision, participated in the performance of key experiments, and made substantial contributions to manuscript revision. Rong Huang, Ruping Wei, Runhui Wang, and Shu Yan performed the pollination experiments. Houyin Deng conducted most of the experiments or data analysis. Shu Yan, Kunjin Han, Juan Han, Meng Ke, and Yuhan Sun conducted most of the experiments. Houyin Deng wrote the initial manuscript. Ye Zhao and Yousry A. El‐Kassaby revised the writing. All authors revised and approved the final manuscript.

## Conflicts of Interest

The authors declare no conflicts of interest.

## Supporting information


**Data S1:** pbi70363‐sup‐0001‐DataS1.zip.

## Data Availability

The RNA‐Seq raw data have been deposited into China National Center for Bioinformation (CNCB) Database (https://www.cncb.ac.cn/) with accession nos.: PRJCA035010.
