# Supplementary material for: ClAPRT3 ‐Mediated Adenine Salvage Pathway Enhances Purine Metabolites to Sustain Seed Vigour During Selfing in Cunninghamia lanceolata
Source: Plant Biotechnol J. 2025 Sep 16;24(2):547–66. doi: 10.1111/pbi.70363 (PMC12906808; doi:10.1111/pbi.70363)
Supplement: Supplementary file 1 — Data S1: pbi70363‐sup‐0001‐DataS1.zip. [file PBI-24-547-s001.zip › pbi70363-sup-0001-Figures.docx]

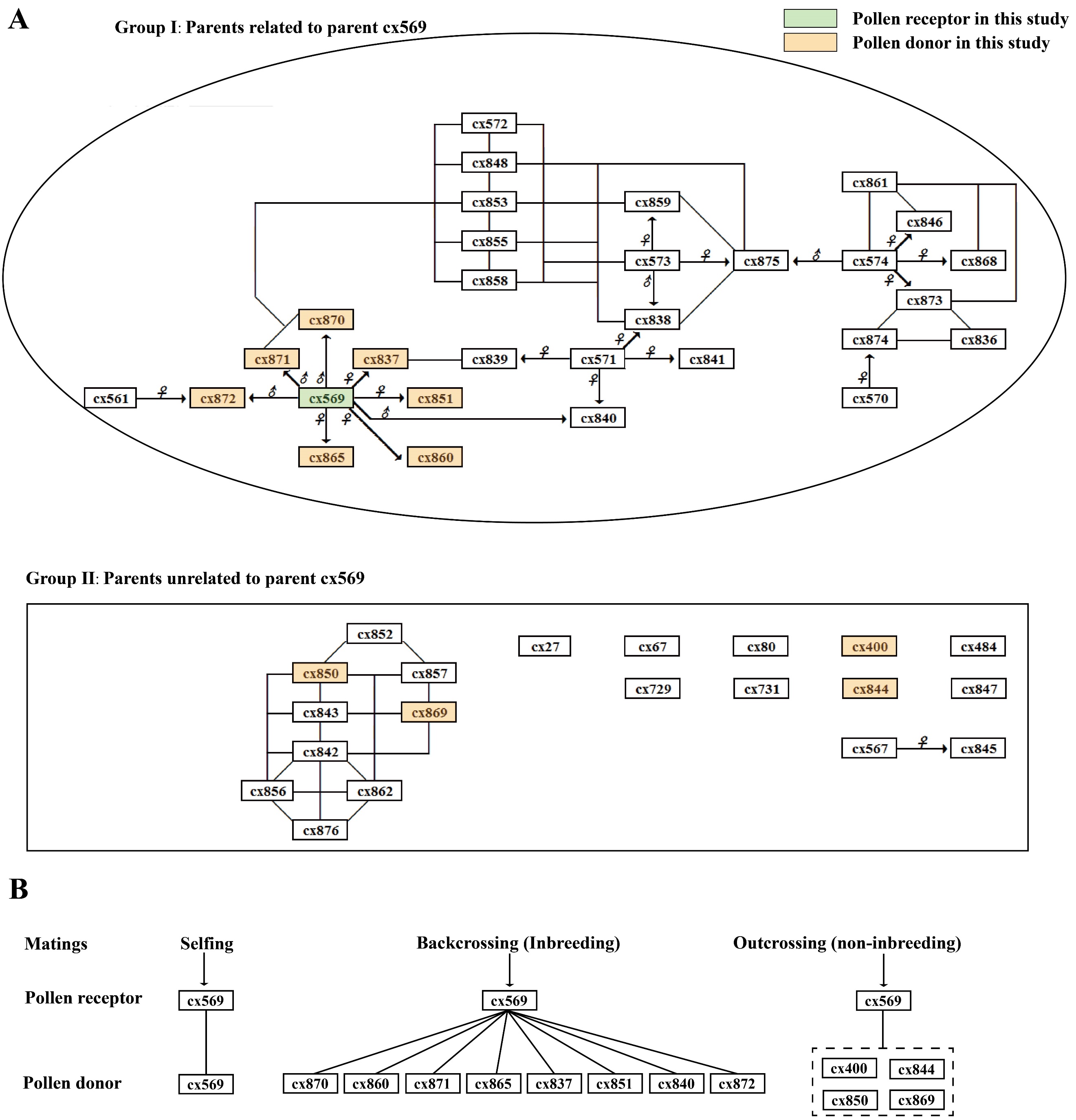


**Figure S1.** Pedigree relationships of pollinated parents and schematic diagram of artificial pollination. (A) The pedigree relationships of the partial Chinese fir parent trees are illustrated using documented pedigree information (**Zeng et al., 2023**). Relationships between parents and offspring are indicated by arrows, while genetically related individuals lacking detailed pedigree records are connected by straight lines. The symbols ♂ and ♀ denote male and female parents, respectively. (B) Schematic diagram of artificial pollination in this study. The self-fertilizing parent cx569 was selected as the pollen receptor, and the pollen produced by the pollen receptor itself was selected for selfing. The first filial generation parents cx837, cx840, cx851, cx860, cx865, cx870, cx871, and cx872 of the self-fertilizing parent cx569 were selected as pollen donors for inbreeding. For non-inbred pollen donors, equal proportions of mixed pollen from cx400, cx844, cx850, and cx869 were randomly selected as the pollen donor.


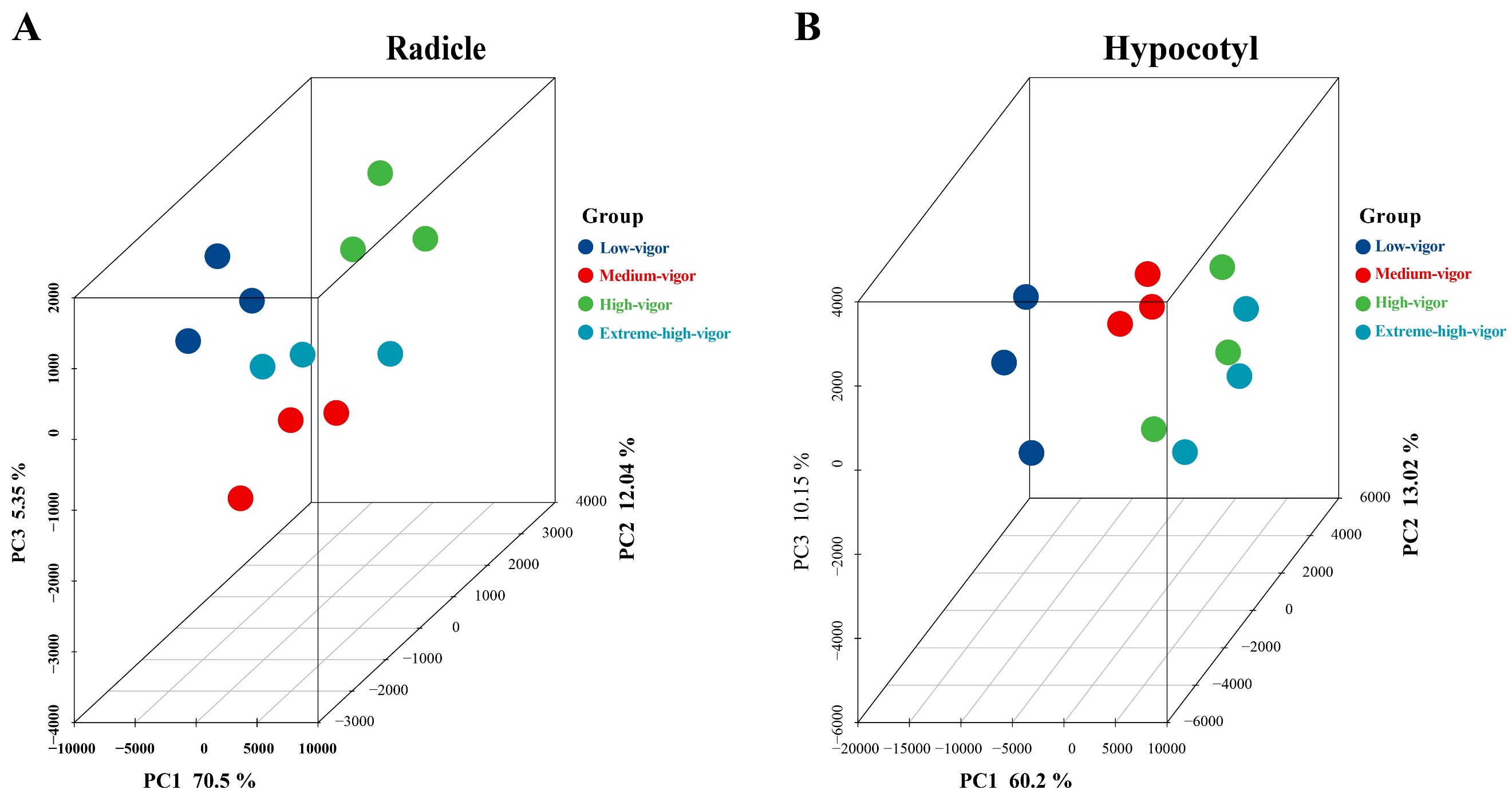


**Figure S2.** Principal component analyses (PCA) were performed using transcriptome data of radicle (A) and hypocotyl (B) of low-vigor (cx569×cx837), medium-vigor (cx569×cx569), high-vigor (cx569×cx840), and extreme-high-vigor (non-inbred) seeds.


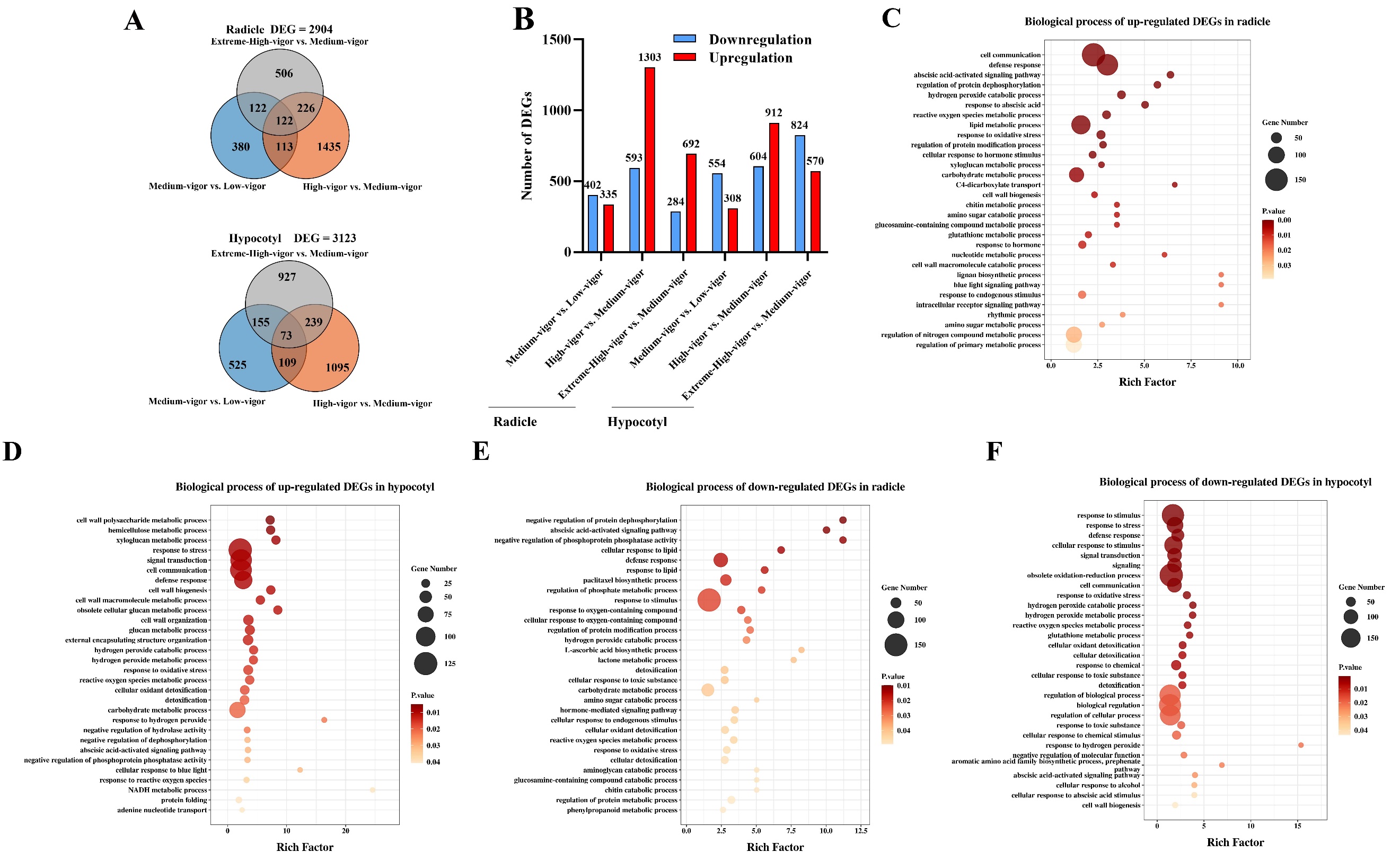


**Figure S3.** Identification of differentially expressed genes (DEGs) and GO enrichment of molecular function. (A) Identification of DEGs across differential comparison groups in the radicle and hypocotyl tissues: medium-vigor vs. low-vigor, high-vigor vs. medium-vigor, and extreme-high-vigor vs. medium-vigor. (B) The number of down- and up-regulated DEGs across differential comparison groups in the radicle and hypocotyl tissues. (C) Biological process of up-regulated DEGs in radicle. (D) Biological process of up-regulated DEGs in hypocotyl. (E) Biological process of down-regulated DEGs in radicle. (F) Biological process of down-regulated DEGs in hypocotyl.


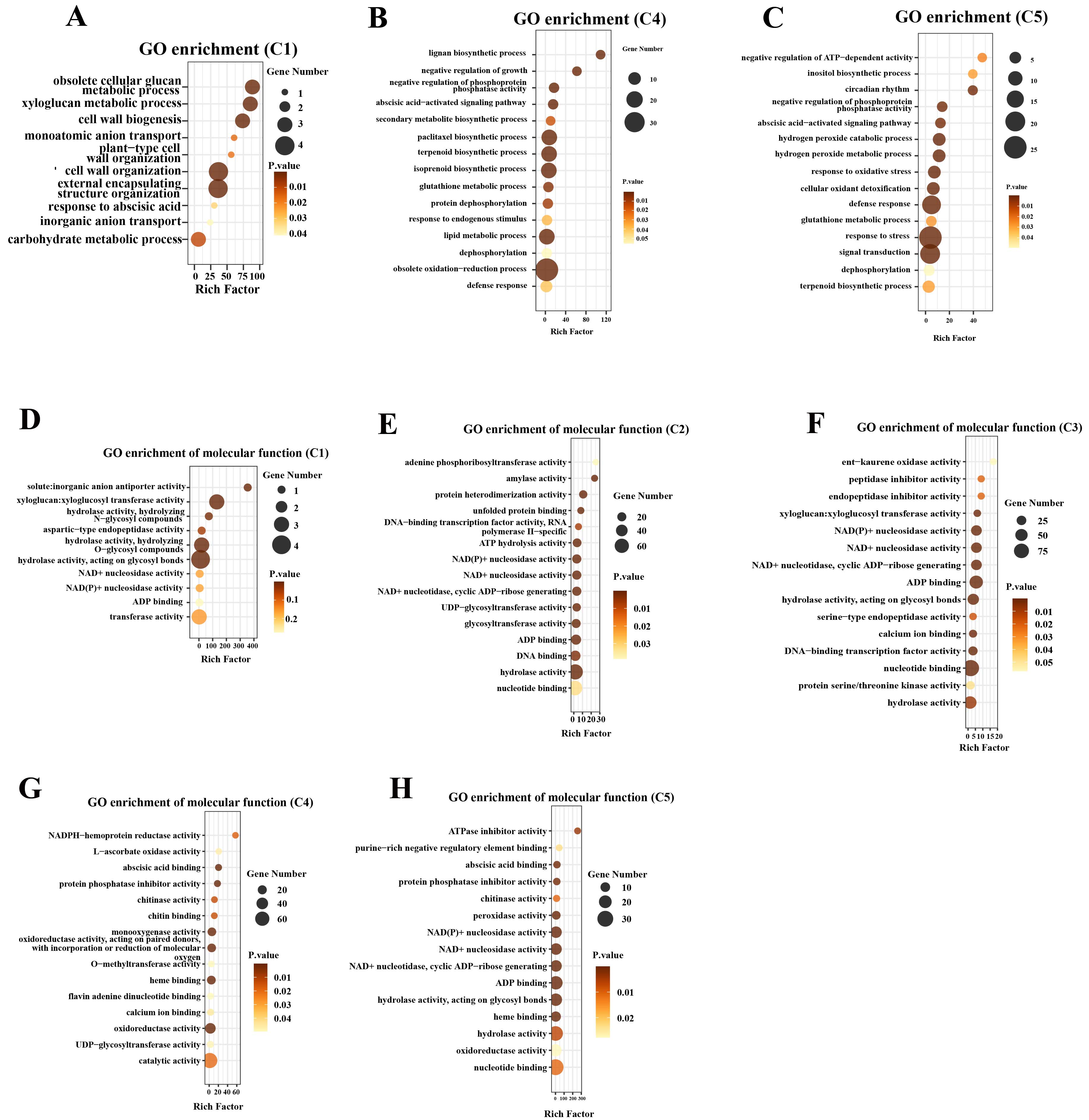


**Figure S4.** Graph (A), (B), and (C) indicate the results of GO enrichment of biological process from C1 (Clustering 1), C4, and C5, respectively. (D), (E), (F), (G), and (H) indicate the results of GO enrichment of molecular function from C1 (Clustering 1), C2, C3, C4, and C5, respectively.


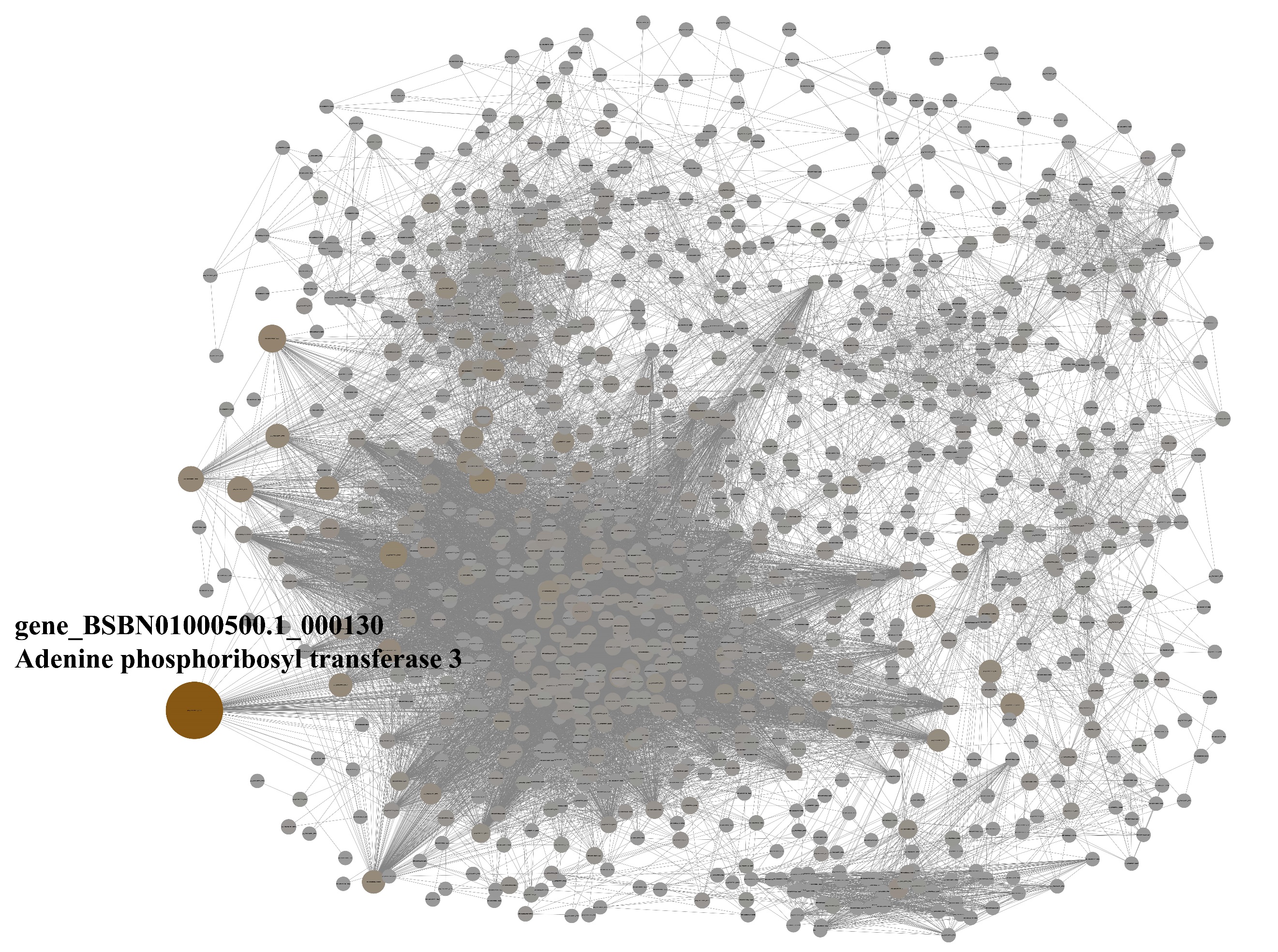


**Figure S5.** Regulatory network generated by the Cytoscape software was based on a Pearson correlation coefficient greater than 0.8 with *P*-value < 0.05. Hub genes were identified using the Stress algorithm in the cytoHubba plug-in of Cytoscape software.


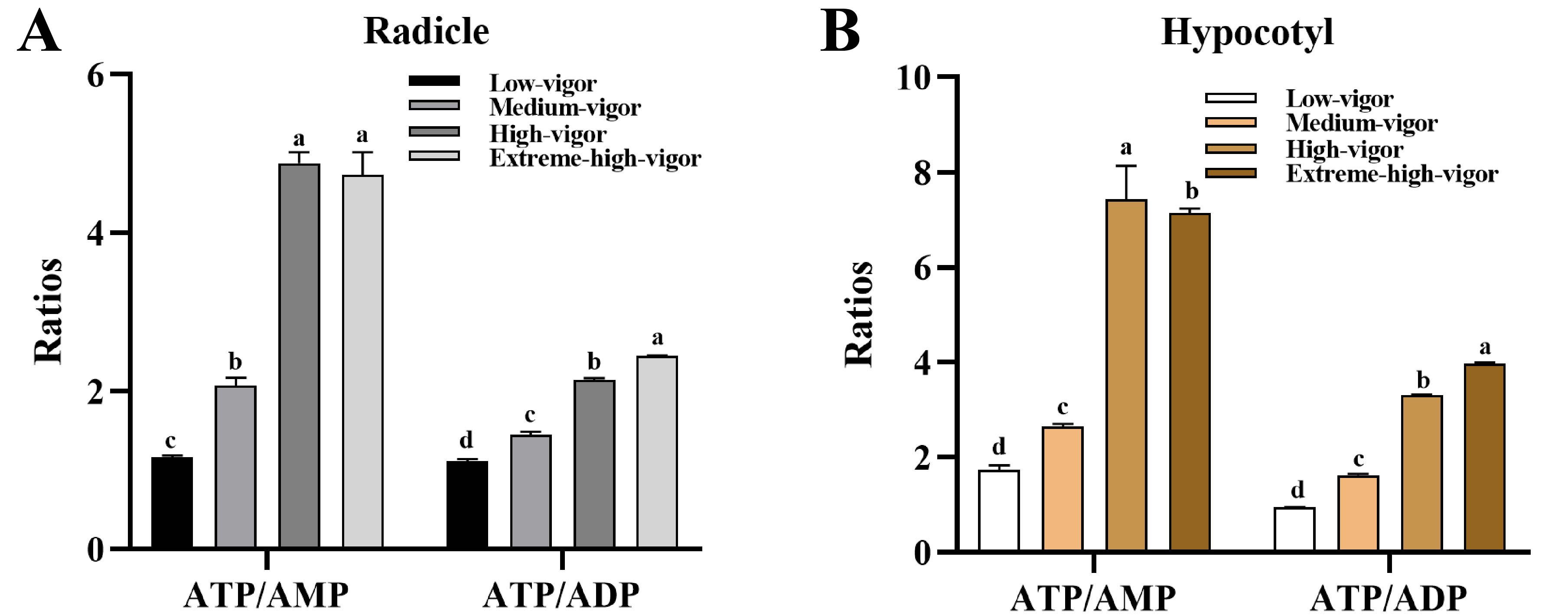


**Figure S6.** The ratios of ATP/AMP and ATP/ADP in radicles and hypocotyl.


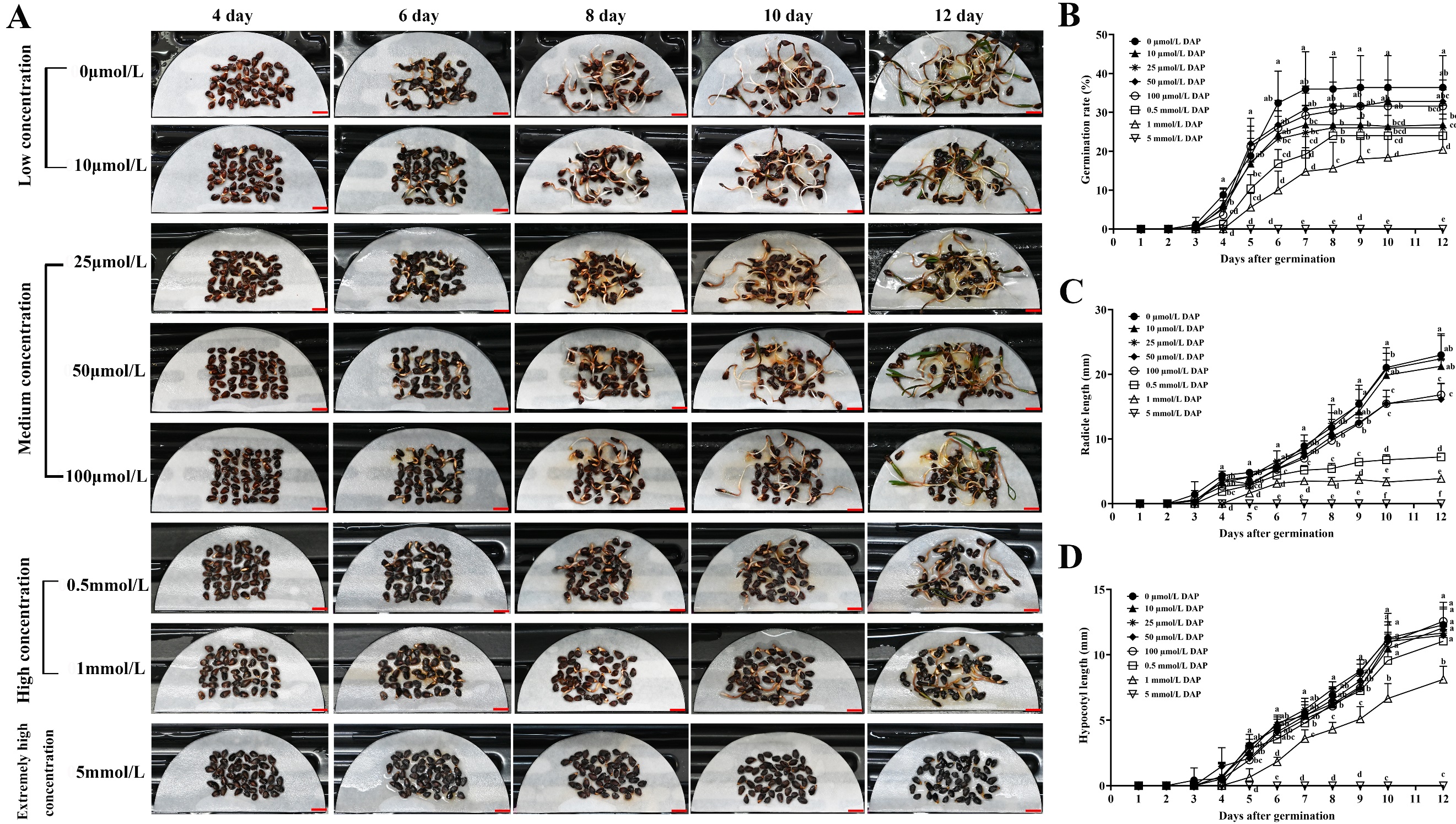


**Figure S7.** Dynamic germination of open-pollinated seeds treated with different concentrations adenine phosphoribosyl transferase (APRT) inhibitor 6-diaminopurine (DAP). (A) Dynamic germination of open-pollinated seeds treated with different concentrations (0 µmol/L, 10 µmol/L, 25 µmol/L, 50 µmol/L, 100 µmol/L, 0.5 mmol/L, 1 mmol/L, and 5 mmol/L) DAP. The red scale represents 1.0 cm. The seed germination (B), radicle length (C) and hypocotyl length (D) of open-pollinated seeds treated with different concentrations DAP.

DAG indicates days after germination. Values represent the mean ± SD. Error bars indicate a standard deviation of five biological replicates with 50 seeds in each replication.


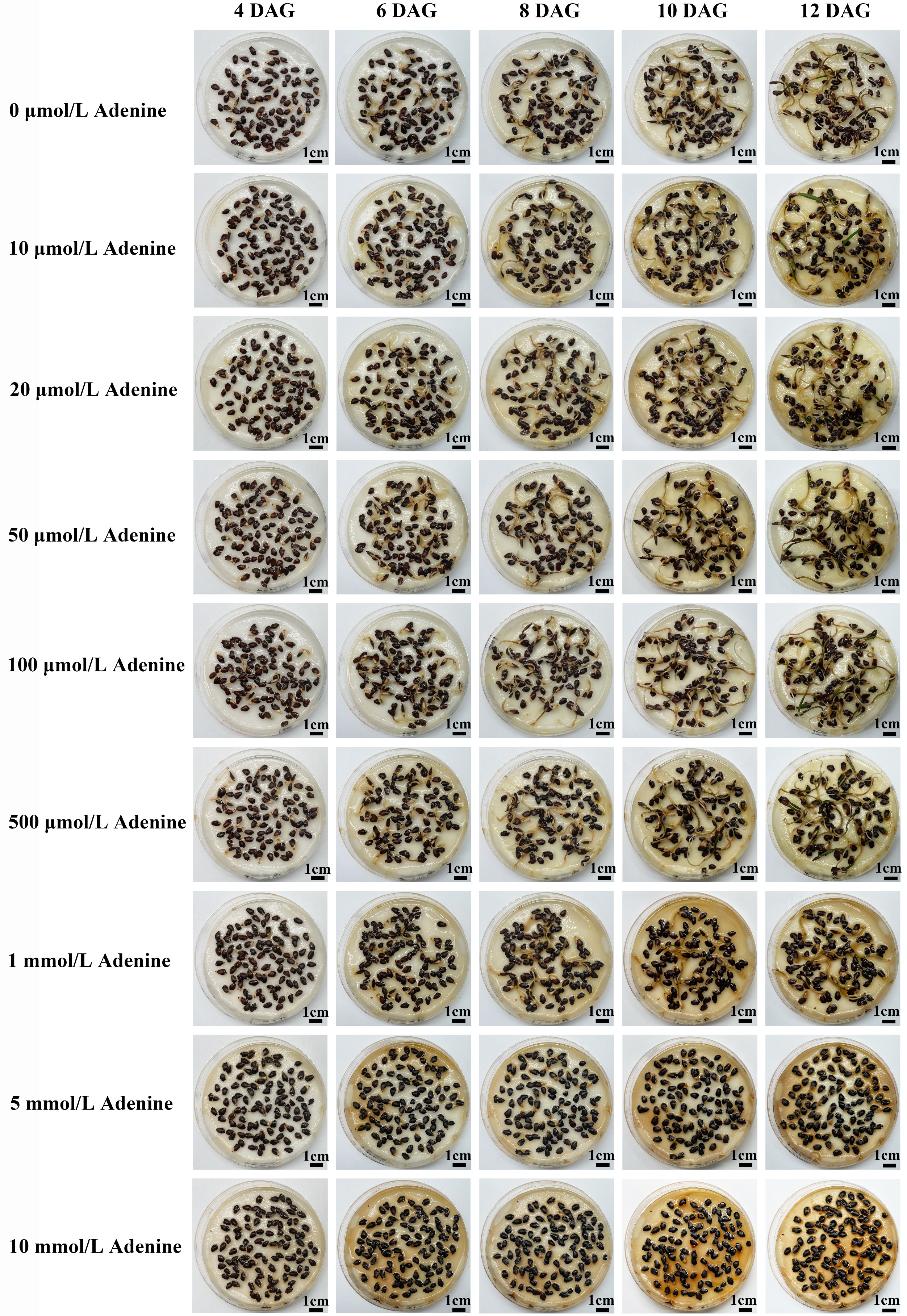


**Figure S8.** Dynamic germination of open-pollinated seeds treated with different concentrations (0 µmol/L, 10 µmol/L, 20 µmol/L, 50 µmol/L, 100 µmol/L, 500 µmol/L, 5 mmol/L, and 10 mmol/L) adenine. DAG indicates days after germination. Each experiment was repeated six times with 100 seeds in each replication. The black scale represents 1.0 cm.


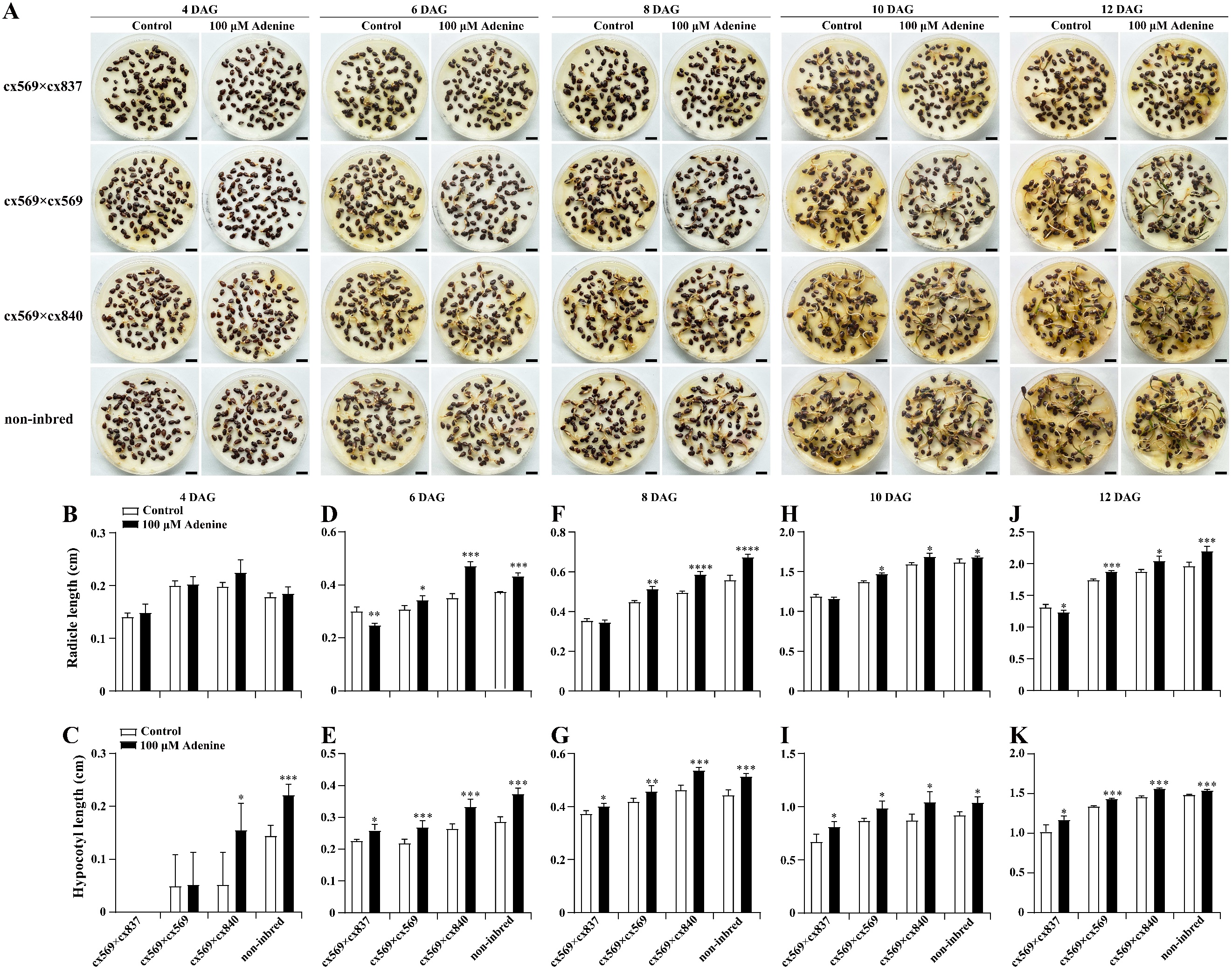


**Figure S9.** Dynamic germination of inbred seeds treated with 100 µmol/L (µM) adenine. (A) Dynamic germination of inbred seeds treated with 1 µM adenine. The black scale represents 1.0 cm. The lengths of radicles at 4 DAG (B), 6 DAG (D), 8 DAG (F), 10 DAG (H), and 14 DAG (J), and the hypocotyls at 4 DAG (C), 6 DAG (E), 8 DAG (G), 10 DAG (I), and 14 DAG (K) of cx569×cx837 (low-vigor), cx569×cx569 (medium-vigor), cx569×cx840 (high-vigor), and non-inbred (extreme-high-vigor) seeds were measured during germination after seed treated with 100 µM adenine. DAG indicates days after germination. Values represent the mean ± SD. Error bars indicate a standard deviation of six biological replicates with 100 seeds in each replication. **P* < 0.05, ***P* < 0.01, ****P* < 0.001, *****P* < 0.0001 (Student’s t test).


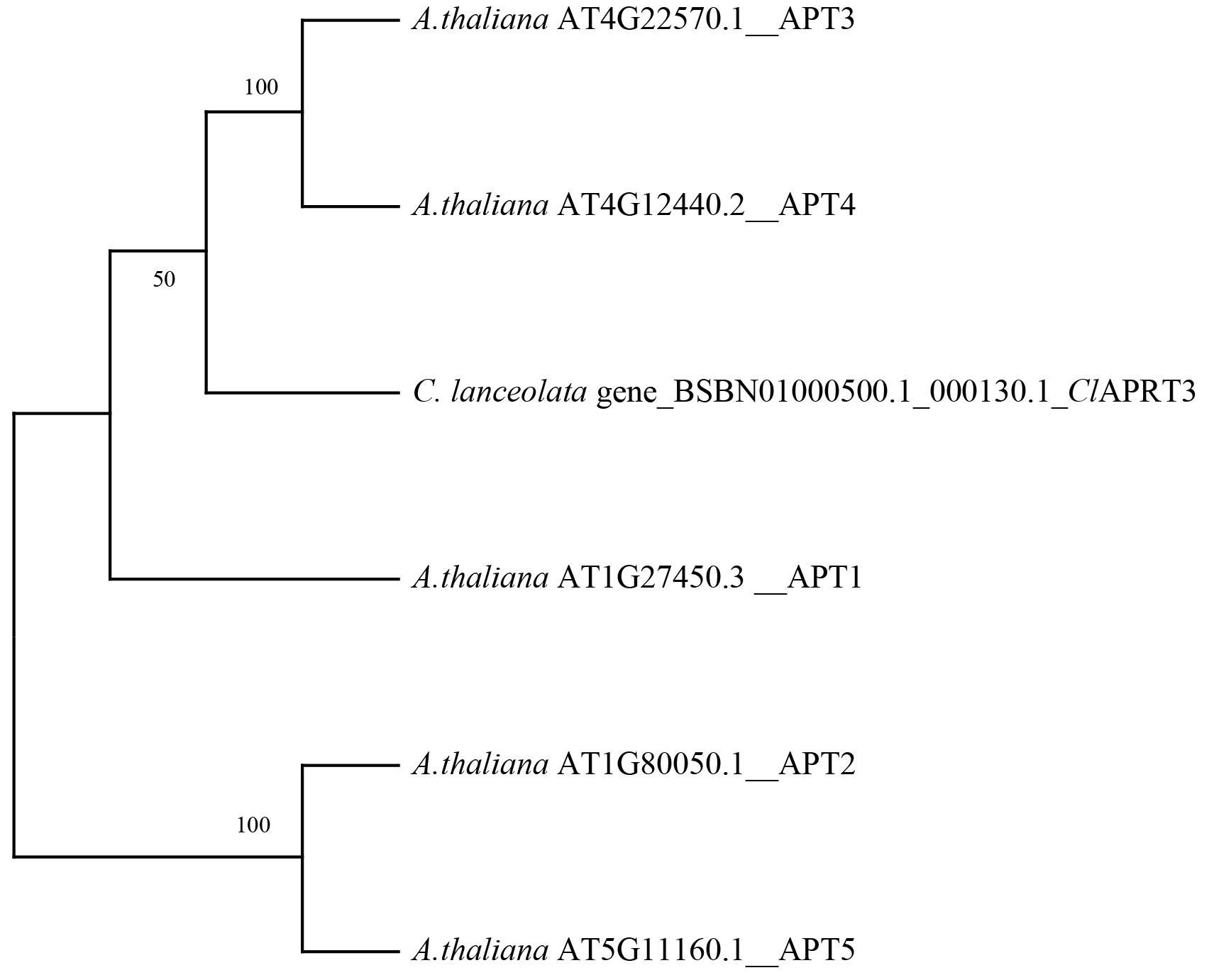


**Figure S10.** The phylogenetic relationships of *ClAPRT3* with other *APRT* family proteins in *Arabidopsis* was inferred using the Neighbor-Joining method. The percentage of replicate trees in which the associated taxa clustered together in the bootstrap test (1000 replicates) are shown next to the branches. Evolutionary analyses were conducted in MEGA7.


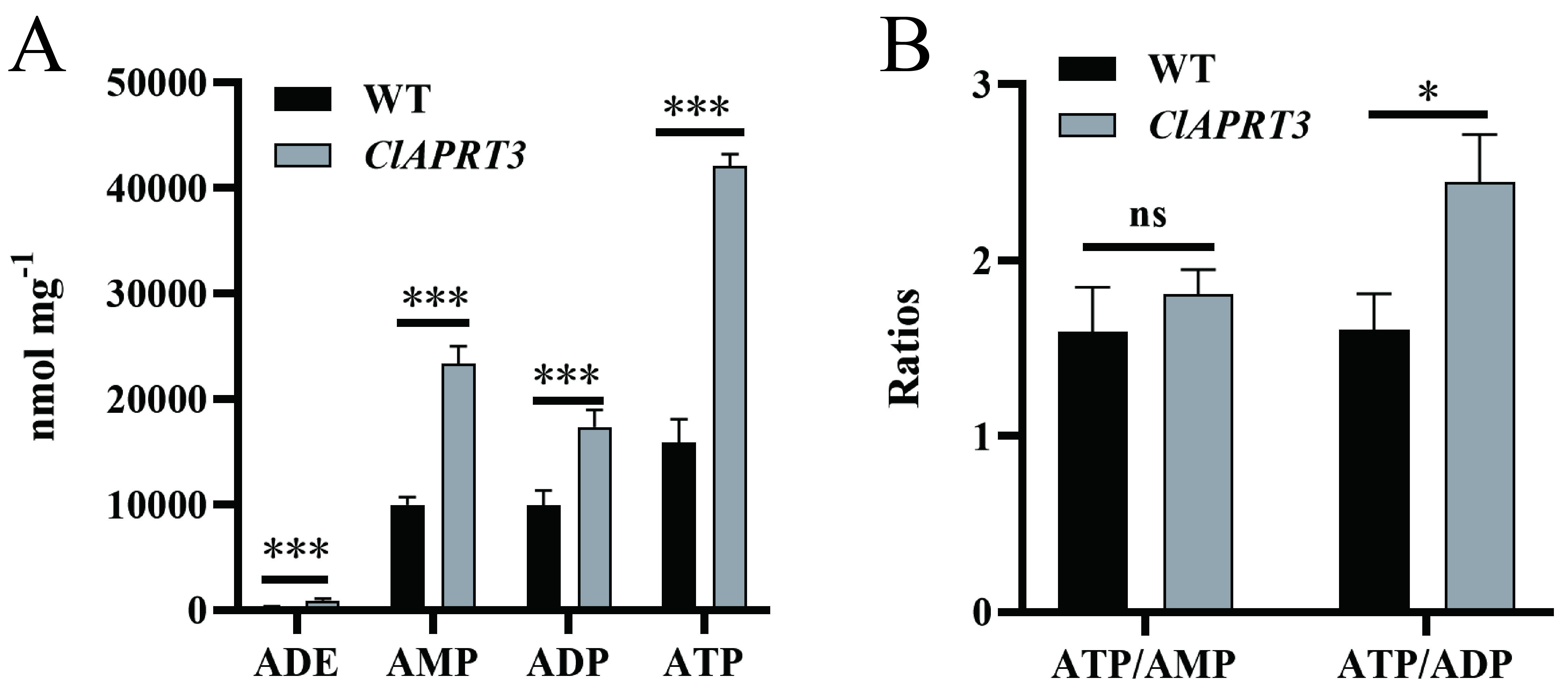


**Figure S11**. Comparison of purine metabolites between wild-type (WT) and *35S::ClAPRT3* *Arabidopsis* seedlings on the 5th day after germination. (A) Levels of ADE, AMP, ADP, and ATP. (B) Ratios of ATP/AMP and ATP/ADP. Error bars indicate means ± SD (n = 3). **P* < 0.05, ****P* < 0.001 (Student’s *t* test). ns indicates no significance.


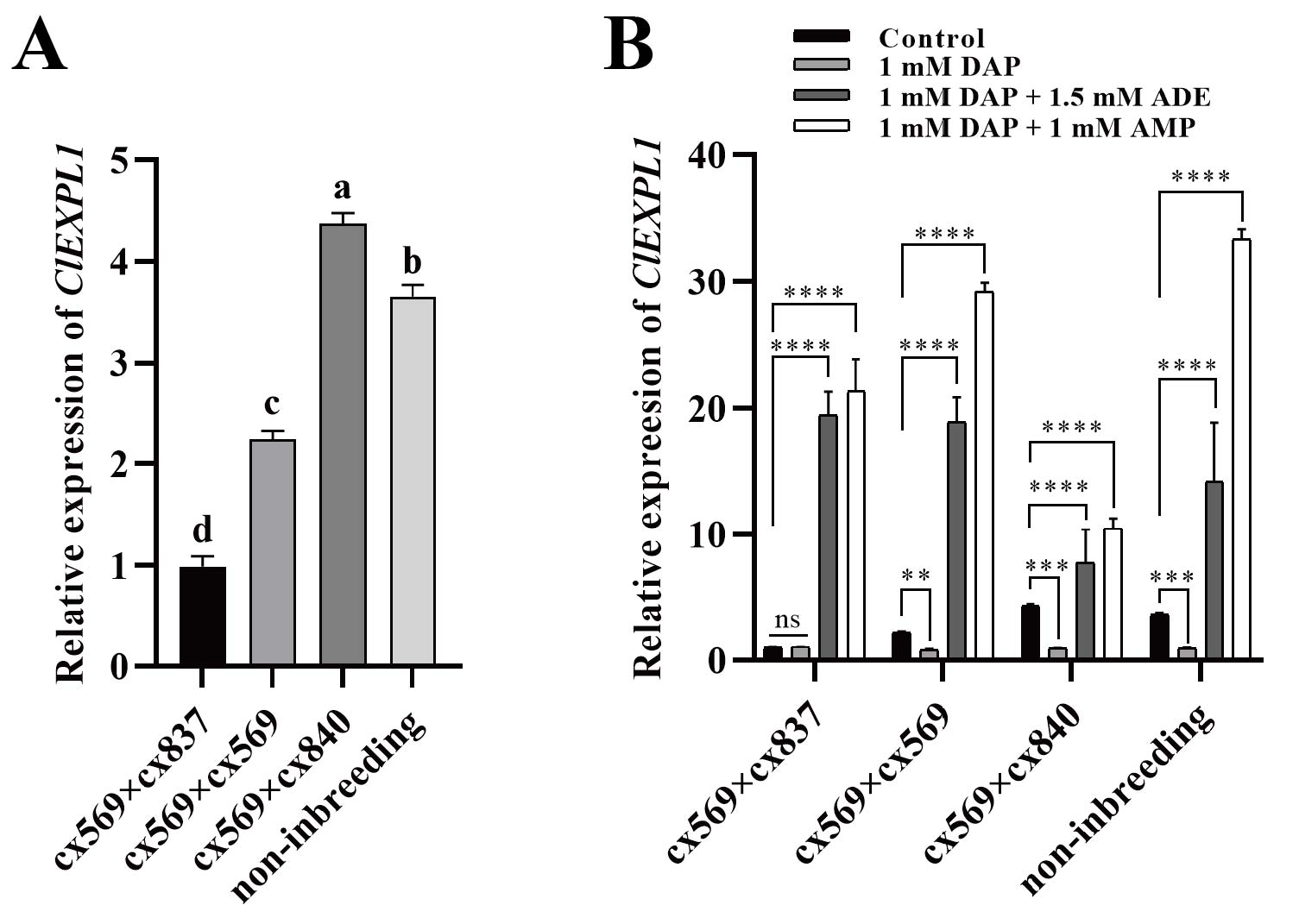


**Figure S12**. Expression of *CLEXPL1* of inbred seeds under treated with DAP, DAP and ADE, and DAP and AMP at 8th DAG. Expression of *CLEXPL1* of inbred and non-inbred seeds under treated with water among inbred seeds (A). Expression of *CLEXPL1* of inbred seeds under treated with 1 mM 6-diaminopurine (DAP), 1 mM DAP and 1.5 mM ADE, and 1 mM DAP and 1 mM AMP (B). DAG, ADE, and AMP indicate days after germination, adenine, and adenosine monophosphate, respectively. Different letters denote statistically significant differences resulting from Duncan’s test following one-way ANOVA. **P* < 0.05, ***P* < 0.01, *****P* < 0.0001 (Student’s t test). Error bars indicate ± SD (n = 3).


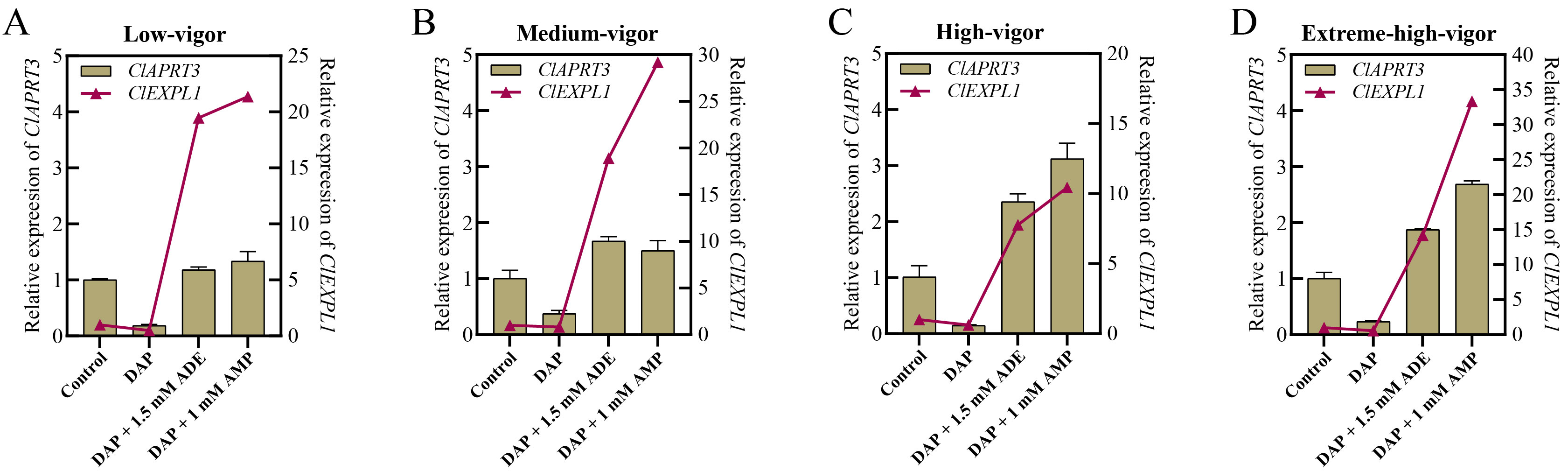


**Figure S13**. Comparison of the expression profiles of *ClAPRT3* and *ClEXPL1* in inbred seeds with low-vigor (A), medium-vigor (B), high-vigor (C), and extreme-high-vigor (D) on day 8 after germination under four treatments: sterile water (control), 1 mM DAP, 1 mM DAP + 1.5 mM ADE, and 1 mM DAP + 1 mM AMP. ADE, adenine; AMP, adenosine-5ʹ-monophosphate; ADP, adenosine diphosphate; ATP, adenosine triphosphate. Error bars indicate ± SD (n = 4).


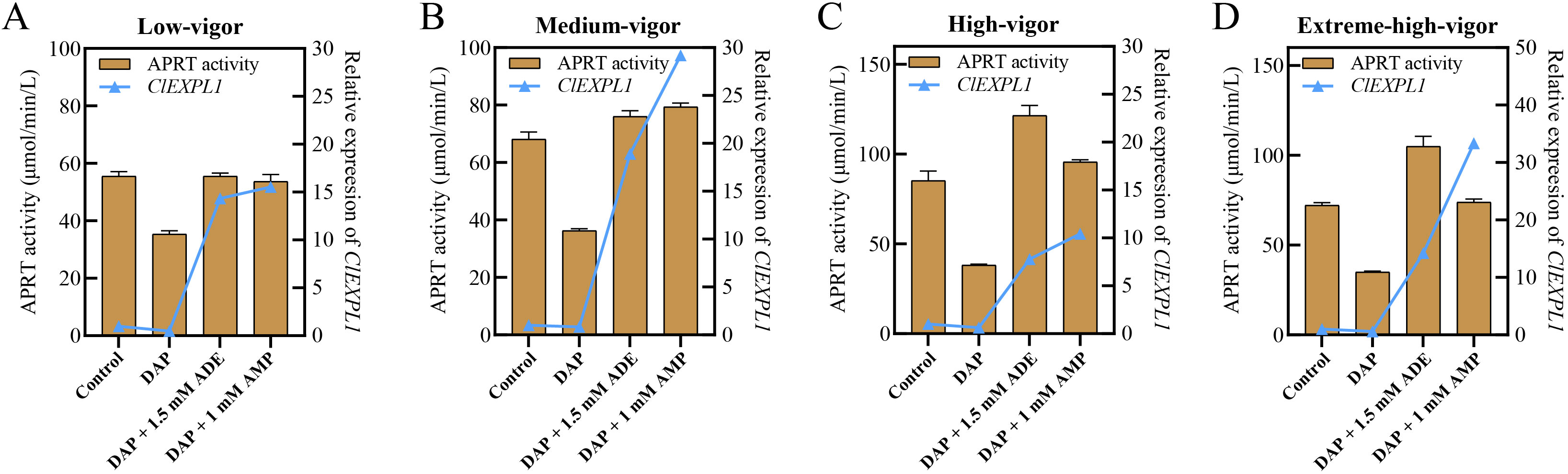


**Figure S14**. Comparison of the APRT activity and the expression profiles of *ClEXPL1* in inbred seeds with low-vigor (A), medium-vigor (B), high-vigor (C), and extreme-high-vigor (D) on day 8 after germination under four treatments: sterile water (control), 1 mM DAP, 1 mM DAP + 1.5 mM ADE, and 1 mM DAP + 1 mM AMP. ADE, adenine; AMP, adenosine-5ʹ-monophosphate; ADP, adenosine diphosphate; ATP, adenosine triphosphate. Error bars indicate ± SD (n = 4).


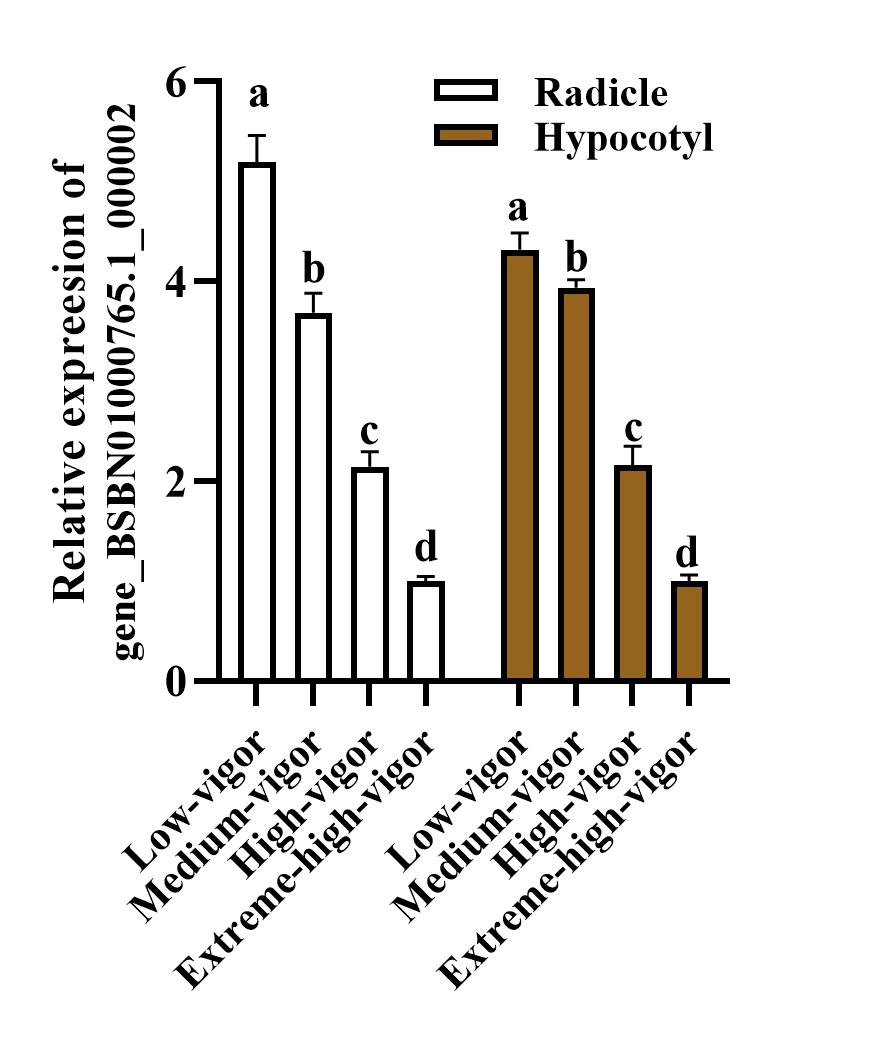


**Figure S15**. Expression of gene_BSBN01000765.1_000002 of inbred and non-inbred seeds under treated with water. Different letters denote statistically significant differences resulting from Duncan’s test following one-way ANOVA. Error bars indicate ± SD (n = 3).


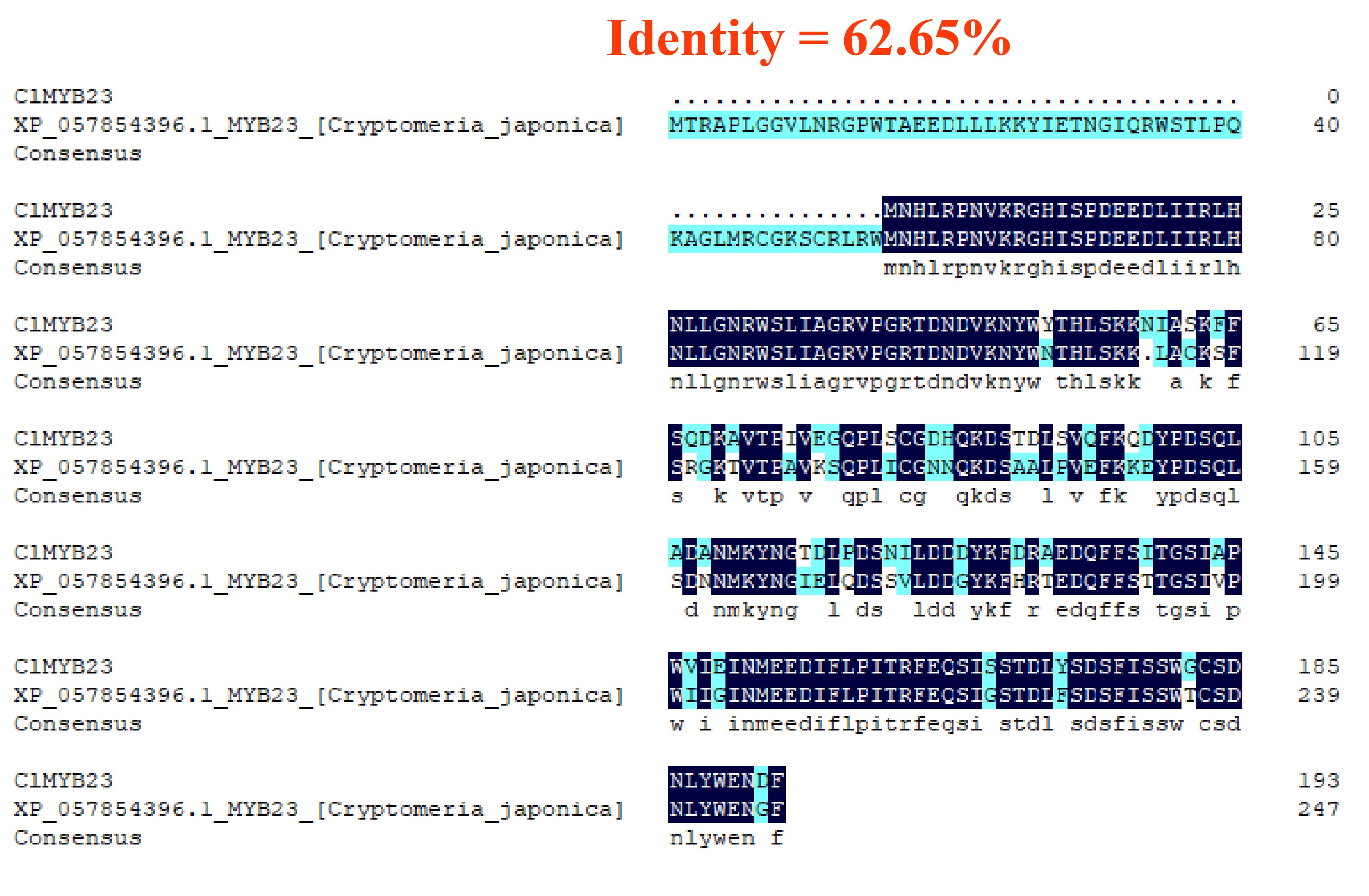


**Figure S16.** Amino acid sequence alignment of *ClMYB23* and closest orthologues from *CjMYB23*.


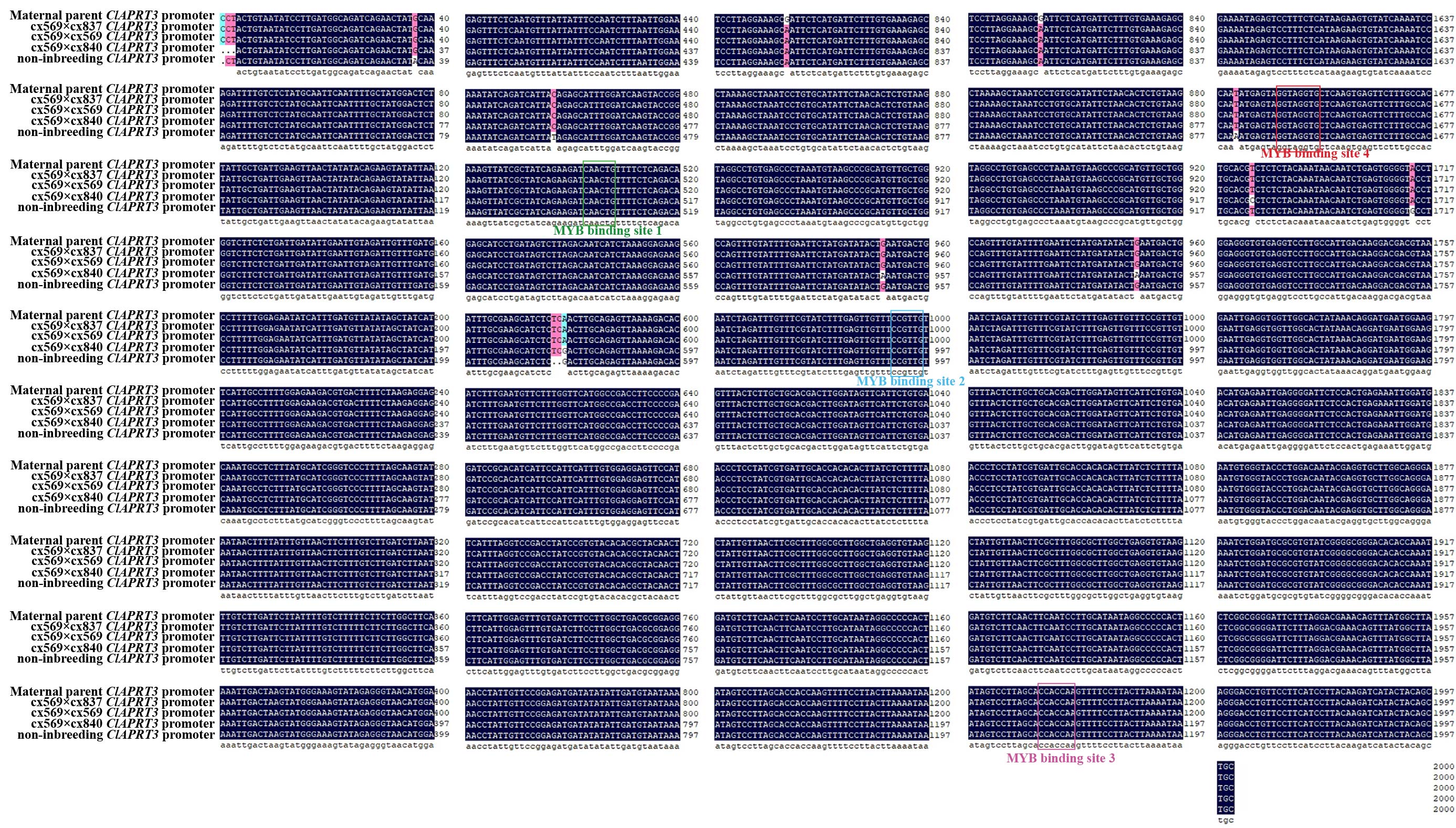


**Figure S17.** Promoter sequence characterization of *ClAPRT3* from different inbred and non-inbred progeny in *Cunninghamia lanceolata*, as well as pollen receptor parent cx569.
